# Geographical specific association between lifestyles and multimorbidity among adults in China

**DOI:** 10.1371/journal.pone.0286401

**Published:** 2023-06-07

**Authors:** Peixi Rong, Yukui Chen, Yusong Dang, Xinyu Duan, Mingxin Yan, Yaling Zhao, Fangyao Chen, Jing Zhou, Duolao Wang, Leilei Pei

**Affiliations:** 1 Department of Epidemiology and Health Statistics, School of Public Health, Xi’an Jiaotong University Health Science Center, Xi’an, Shaanxi, P.R. China; 2 Department of Pediatrics, The Second Affiliated Hospital of Xi’an Jiaotong University, Xi’an, Shaanxi, P.R. China; 3 Biostatistics Unit, Department of Clinical Sciences, Liverpool School of Tropical Medicine, Liverpool, United Kingdom; University of International Business & Economics, CHINA

## Abstract

The relationship between lifestyles and multimorbidity is well established, but previous studies have often neglected the role of spatial heterogeneity. Thus, this study is the first to explore this association in Chinese adults from a spatial perspective using a geographically weighted logistic regression (GWLR) model and describe the geographical characteristics across different regions. According to 2018 China Health and Retirement Longitudinal Study (CHARLS) database, a total of 7101 subjects were finally included, with 124 prefecture-level administrative regions in China. Non-spatial and GWLR model were used for analysis, and gender stratification analysis was also performed. Data were visualized through ArcGIS 10.7. The results showed that a total prevalence of approximately 5.13% of multimorbidity, and among participants with multimorbidity, the separate prevalence of hypertension, diabetes or high blood sugar, heart disease, and stroke were 4.45%, 2.32%, 3.02%, and 1.41%, respectively. The GWLR model indicated that current (OR: 1.202–1.220) and former smokers (OR: 1.168–1.206) may be important risk factors for multimorbidity in adults, especially in north and west among male. Past drinkers (OR: 1.233–1.240), especially in eastern China, contribute to the development of the multimorbidity in men but not in women. Vigorous-intensity activities (OR: 0.761–0.799) were negatively associated with multimorbidity in the west, with no gender difference. Depression (OR: 1.266–1.293) appeared to increase the risk for multimorbidity, with the weakest effects in central China and no gender difference. There was an interaction between light activities and gender (*P* = 0.024). The prevalence of multimorbidity differed across various areas of the province. The role of geographical variations in lifestyles and multimorbidity may provide valuable information for developing site-specific intervention strategies.

## Introduction

Multimorbidity, defined as the coexistence of two or more chronic non-communicable diseases in a single patient, tends to increase gradually with age and is more prevalent in adults [1]. Researches have demonstrated that multimorbidity can result in poor physical function and quality of life, increased healthcare costs and disease burden, and higher disability and mortality risks in adults. As a result, multimorbidity has become a significant public health challenge worldwide [2–5]. In recent years, a growing body of research has revealed that unhealthy lifestyles are closely related to the occurrence of multimorbidity [6–8]. For example, it was found that participants with lower levels of physical activity are more likely to develop multiple chronic diseases than those who engage in regular physical activity [9]. Some evidences showed that the incidence rate of multimorbidity increased more rapidly with age among participants who smoked and drank excessively [10].

Studies have estimated the prevalence of multimorbidity in Europe (29.7%), Indonesia (35.7%), the United States (21%), and Australia (25.5%), demonstrating that there are significant differences in the prevalence between different geographic regions [3, 11–13]. While environmental and behavioral components play a crucial role in the etiological progression of multimorbidity, the association between lifestyles and multimorbidity among adults hasn’t been consistent across regions. For example, smoking and consuming alcohol could increase the prevalence of multimorbidity among Indian women [18]. But, smoking has been shown to have a negative correlation with multimorbidity in South Africa, and alcohol consumption was not identified as a risk factor for cardiometabolic diseases in South Asians [19, 20]. Additionally, a Brazilian study showed no significant association between long sleep duration and multimorbidity in individuals [21], while a study in China revealed that participants with shorter (<7 h) and longer (>9 h) sleep duration had a higher prevalence of multimorbidity [14]. Because of socio-cultural and geographical reasons, lifestyle-related factors may differ and population-specific in each area. What remains unknown is how factors that influence multimorbidity vary across regions. Previous studies have mostly used traditional non-spatial methods that ignored the important role of spatial heterogeneity in the correlation between lifestyles and multimorbidity. Thus, cross-regional comparisons of the mechanisms by which lifestyles impact cardiometabolic multimorbidity are still needed.

The geographically weighted logistic regression (GWLR) model provides a powerful tool for examining local variations and visualizing spatial heterogeneity, enabling clear demonstration of the differences in the association of lifestyles with multimorbidity among adults between regions. Therefore, this study employed the GWLR model to generate local coefficients to account for geographic differences in the relationship between lifestyles and multimorbidity among adults based on China Health and Retirement Longitudinal Study (CHARLS) database. Thus, the objectives of the current study are twofold: (1) to describe the geographic distribution of multimorbidity and lifestyles across different regions, (2) and to explore the spatially varying relationship between them among adults.

## Materials and methods

### Data collection

The data in this study were collected from the CHARLS, which is a survey of Chinese residents aged 45 and older conducted by Peking University. The baseline national assessment of CHARLS was carried out in 2011, and residents were selected from 150 counties and 450 communities (villages) of 28 provinces in China using a stratified multi-stage probability-proportional-to-size random sampling strategy [15]. Wave 2 for CHARLS was conducted in 2013, wave 3 in 2015, and wave 4 in 2018. All data and details are publicly available and can be accessed at: https://charls.charlsdata.com/pages/data/111/zh-cn.html The CHARLS has been approved by the Biomedical Ethics Review Committee of Peking University. All the data used in our study was public from the official website of the government and didn’t involve copyright issues. To protect the privacy of the individuals, all data analyzed were anonymized in occasion of data use, processing, sharing and interaction. None of the study personnel could see the personal information of individuals. More detailed descriptions of the objectives and methods have been reported elsewhere [15].

The CHARLS 2018 dataset of 19817 cases was adopted for the current analysis. A total of 12716 participants were excluded due to missing data, including multimorbidity (7117), age (450), smoking (7446), drinking (87), sleep duration (18), physical activities (84), and depression (1664). The final sample size for analysis was 7,101 cases.

The outcome variable of the study was multimorbidity, defined as the co-occurrence of at least two chronic diseases, including hypertension, diabetes or high blood sugar, heart disease (heart attack, coronary heart disease, angina, congestive heart failure, or other heart problems), and stroke. Sociodemographic characteristics containing age and gender were used as adjustment variables. And lifestyles included smoking, drinking, sleep duration, physical activities, and depression. More detailed definitions and classifications of variables were presented in S1 File.

### Statistical analysis

The analysis procedures were presented as follows. First, the geographic distributions of lifestyles and multimorbidity among the participants were depicted on a China map using ArcGIS 10.7 software. The geographic coordinates (latitude/longitude) of the prefecture-level cities served as the basic geographic units for this study. The geographic information of the surveyed cities was obtained using the vector map of China provided by the National Geospatial Information Public Service Platform (http://www.tianditu.com/).

Second, the non-spatial logistic regression model was applied to investigate the association between lifestyles and multimorbidity among adults while controlling for confounders through IBM SPSS Statistics 26. The interactions were also tested by comparing models with and without a cross-product term between the risk factors and gender for logistic regression. Third, the GWLR model was introduced to evaluate the geographic differences in the relationship after adjusting for all potential confounders, and local average estimates for each individual were displayed on a map using ArcGIS 10.7 software. Fourth, we repeated the non-spatial logistic regression and GWLR model analyses for participants with different genders.

Age was set as a dummy variable, and the 45–54 years old group was used as the reference group. Gender was coded as male = 0 and female = 1. Smoking, drinking, and sleep duration were also transformed into dummy variables. For smoking: current smokers (1 = yes, 0 = no) and former smokers (1 = yes, 0 = no), with non-smokers serving as the reference group. Similarly, for drinking: current drinkers (1 = yes, 0 = no) and past drinkers (1 = yes, 0 = no), with non-drinkers serving as the reference group. Sleep duration: <6 h (1 = yes, 0 = no) and >8 h (1 = yes, 0 = no), with the reference group being those who reported sleeping 6–8 h.

The GWLR model was expressed as the following equation [16].


$$log(\frac{P(y_{i}=1)}{1-P(y_{i}=1)})=\beta_{0i}(u_{i},v_{i})+\sum_{j=1}^{k}\left(\beta_{ij}(u_{i},v_{i})x_{ij}\right)$$

The equation hypothesizes that *y_i_* (dependent variables) was any chronic multimorbidity for each individual i, *x_ij_* representes a set of independent variables (j = 1,…, k) for the individual i, (*u_i_, v_i_*) was regarded as the x-y coordinates of the individual i, *β_ij_* (regression coefficient) was the estimated effect of independent variable j for the individual i. In the GWLR model, the OR corresponds to the unit change of the variable, which is exponentiation of the regression coefficients. The value was used to intuitively reflect the geographical differences in the relationship between lifestyles and multimorbidity.

In the modeling process, an iterative reweighted least squares approach was used to estimate GWLR equation. A distance-based weighting scheme was adopted to assign weights to each prefecture-level city based on the observations for nearby prefecture-level cities. The kernel type and function for geographical weighting to estimate local coefficients and their bandwidth size was adaptive bisquare. The modified Akaike Information Criterion (AICc) was chosen as the golden search method to determine the bandwidth in the adaptive kernel. In this study, MGWR 2.2 software (https://sgsup.asu.edu/sparc/mgwr) was employed for GWLR analysis. A two-tailed P<0.05 was regarded as statistically significant.

## Results

### Baseline characteristics of the participants

In this present study, a total of 7101 subjects from 124 prefecture-level administrative regions in China were sampled. The age of the subjects ranged from 45 to 97 years old, with a mean age of 58.74 years old. The proportion of females was higher than males (76.92% vs 23.08%, P<0.001). Of the participants, the percentage of non-smokers and non-drinkers was 91.35% and 80.69%, respectively. Approximately 58.57% of the participants slept 6–8 hours. Most of the participants engaged in moderate (56.50%) and light activities (84.14%), while the participation with vigorous-intensity activities (33.18%) was lower. Moreover, 43.74% of the study population experienced depressive symptoms. More detailed characteristics of participants were presented in Table 1.

**Table 1:** Characteristics of enrolled adult participants by gender.

Variable	Total	Male	Female	χ^2^	*P* ^a^
	n(%)	n(%)	n(%)		
***Sociodemographic characteristics***					
**Age**					
45–54	2910(41.00)	658(40.15)	2252(41.23)	13.912	0.003
55–64	2293(32.30)	494(30.14)	1799(32.94)		
65–74	1410(19.86)	347(21.17)	1063(19.46)		
≥75	488(6.87)	140(8.54)	348(6.37)		
***Lifestyles***					
**Smoking**					
Non-smoker	6487(91.35)	1081(65.95)	5406(98.97)	1740.165	<0.001
Current smoker	355(5.00)	323(19.71)	32(0.59)		
Former smoker	259(3.65)	235(14.34)	24(0.44)		
**Drinking**					
Non-drinker	5730(80.69)	780(47.59)	4950(90.63)	1503.911	<0.001
Current drinker	1183(16.66)	729(44.48)	454(8.31)		
Past drinker	188(2.65)	130(7.93)	58(1.06)		
**Sleep Duration**					
<6h	2307(32.49)	549(33.50)	1758(32.19)	2.310	0.315
6-8h	4159(58.57)	957(58.39)	3202(58.62)		
>8h	635(8.94)	133(8.11)	502(9.19)		
**Vigorous-Intensity Activity**					
No	4745(66.82)	1013(61.81)	3732(68.33)	24.178	<0.001
Yes	2356(33.82)	626(38.19)	1730(31.67)		
**Moderate Activity**					
No	3089(43.50)	865(52.78)	2224(40.72)	74.585	<0.001
Yes	4012(56.50)	774(47.22)	3238(59.28)		
**Light activities**					
No	1126(15.86)	256(15.62)	870(15.93)	0.090	0.764
Yes	5975(84.14)	1383(84.38)	4592(84.07)		
**Depression**					
No	3995(56.26)	1102(67.24)	2893(52.97)	104.325	<0.001
Yes	3106(43.74)	537(32.76)	2569(47.03)		

^a^ Statistically significant between gender were derived from the **χ**^**2**^ test for categorical variables (*P*<0.05).

**Fig 1:**
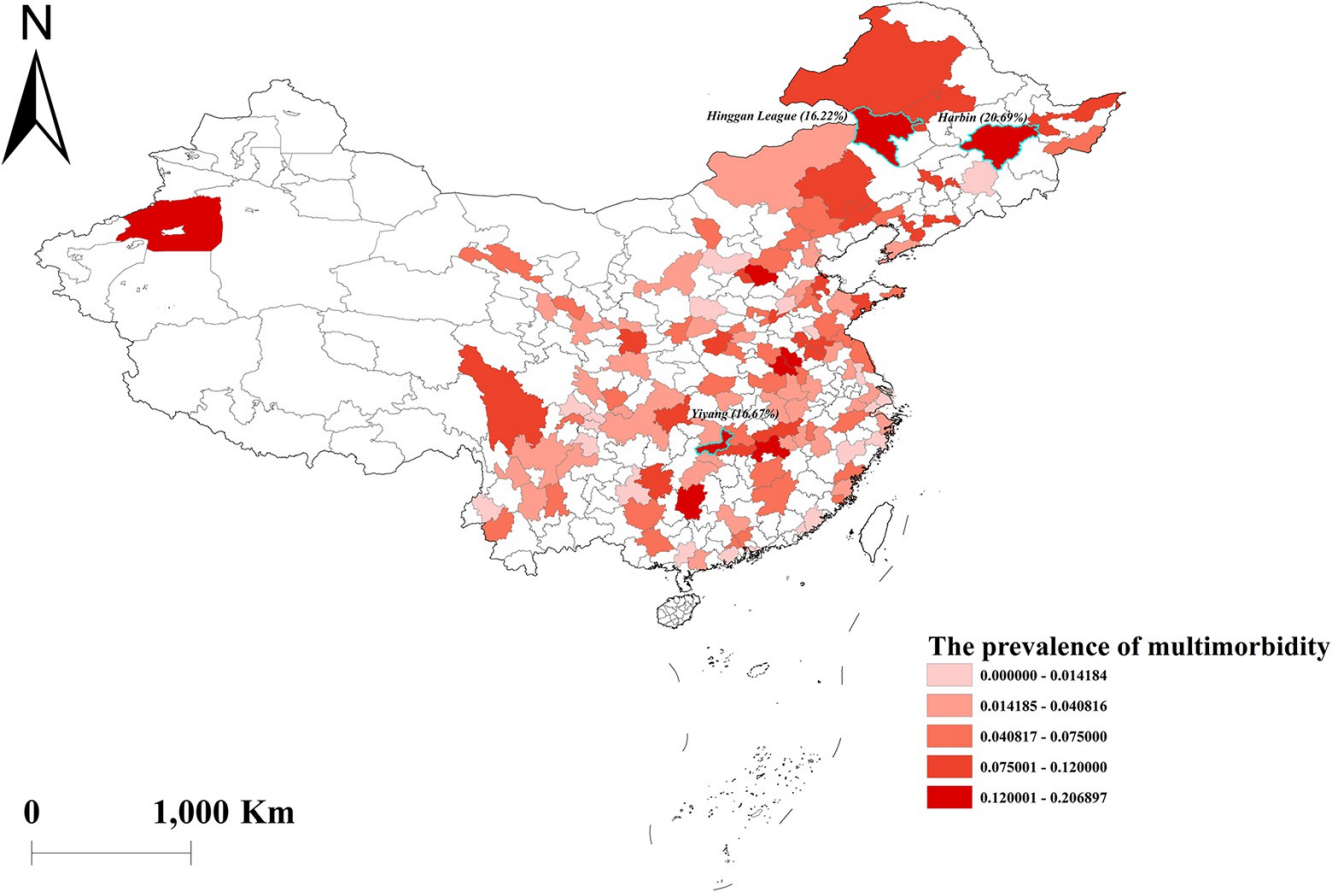
Geographical distribution of the prevalence of multimorbidity in China. Note: The three cities with the highest prevalence are highlighted in Fig 1 with fluorescence labeling: Harbin (20.69%), Yiyang (16.67%), and Hinggan League (16.22%).

The multimorbidity in this study consisted of at least two of the following diseases: hypertension, diabetes or high blood sugar, heart disease, and stroke. Of the 7101 study subjects, approximately 5.13% suffered from multimorbidity. Among those with multimorbidity, the separate prevalence of hypertension, diabetes or high blood sugar, heart disease, and stroke were 4.4%, 2.3%, 3.0%, and 1.4%, respectively (Table 2). The geographic distribution of multimorbidity was shown in Fig 1, with the three cities having the highest prevalence being Harbin (20.69%), Yiyang (16.67%), and Hinggan League (16.22%).

**Table 2:** The prevalence of individual’s diseases among participants with multimorbidity and the total population.

Variable	Comorbidity		Total n(%)
	No(%)	Yes(%)	
**Hypertension**			
No(%)	6033(89.55)	48(13.19)	6081(85.64)
Yes(%)	704(10.45)	316(86.81)	1020(14.36)
**Diabetes or High Blood Sugar**			
No(%)	6524(96.84)	199(54.67)	6723(94.68)
Yes(%)	213(3.16)	165(45.33)	378(5.32)
**Heart Disease** ^**a**^			
No(%)	6445(95.67)	149(40.93)	6594(92.86)
Yes(%)	292(4.33)	215(59.07)	507(7.14)
**Stroke**			
No(%)	6648(98.68)	264(72.53)	6912(97.34)
Yes(%)	89(1.32)	100(27.47)	189(2.66)
**Total**	6737(94.87)	364(5.13)	7101(100.00)

^a^ Heart disease include heart attack, coronary heart disease, angina, congestive heart failure, or other heart problems

### Non-spatial logistic regression

Table 3 summarized the significant correlations between lifestyles and multimorbidity in adults using the logistic regression model adjusting for other possible confounders. Compared to adults aged 45–54 years old, the prevalence of multimorbidity was higher among those aged ≥ 55 years old, with an ascending trend with aging. Current and former smokers (OR: 2.316, 95%CI: 1.499–3.579 and OR: 2.296, 95%CI: 1.444–3.652, respectively) were more likely to suffer from multimorbidity compared to non-smokers. Past drinkers were also more likely to develop multimorbidity than non-drinkers (OR: 3.665, 95%CI: 2.401–5.594). Vigorous-intensity activities were negatively associated with the prevalence of multimorbidity (OR: 0.613, 95%CI: 0.470–0.798). Moreover, depression was found to increase the risk of multimorbidity (OR: 1.721, 95%CI: 1.378–2.149). However, no significant association was found between sleep duration, moderate and light activities, and multimorbidity in the study areas.

**Table 3:** The association between lifestyles and multimorbidity among participants using global logistic model.

Variable	β(SE)	OR	OR(95% CI)		*P*
			lower	upper	
**Intercept**	-3.654(0.242)	0.026			<0.001
**Age (ref = 45–54)**					
55–64	0.426(0.150)	1.532	1.143	2.054	0.004
65–74	1.053(0.148)	2.868	2.147	3.830	<0.001
≥75	0.988(0.196)	2.687	1.830	3.945	<0.001
**Gender (ref = Male)**					
Female	-0.004(.162)	0.996	0.724	1.368	0.978
**Smoking (ref = Non-smoker)**					
Current smoker	0.840(0.222)	2.316	1.499	3.579	<0.001
Former smoker	0.831(0.237)	2.296	1.444	3.652	<0.001
**Drinking (ref = Non-drinker)**					
Current drinker	-0.057(0.168)	0.944	0.679	1.313	0.734
Past drinker	1.299(0.216)	3.665	2.401	5.594	<0.001
**Sleep Duration (ref = 6-8h)**					
<6h	0.015(0.121)	1.167	0.985	0.777	1.247
>8h	-0.026(0.199)	0.679	0.974	0.659	1.440
**Vigorous-Intensity Activity (ref = No)**					
Yes	-0.490(0.135)	0.613	0.470	0.798	<0.001
**Moderate Activity (ref = No)**					
Yes	-0.156(0.115)	0.856	0.683	1.072	0.176
**Light Activities (ref = No)**					
Yes	0.132(0.150)	1.141	0.850	1.532	0.378
**Depression (ref = No)**					
Yes	0.543(0.113)	1.721	1.378	2.149	<0.001

SE: standard error; OR: odds ratio is adjusted OR; CI: confidence interval

According to different gender, the associations between lifestyles and multimorbidity were broadly consistent with our main findings (Table 4). However, the positive association between current smokers and former smokers and the risk for multimorbidity was found in male, and the effect of gender on appreciable modification of smoking was not significant. Light activities were associated with a higher risk of multimorbidity only in men (OR: 2.043, 95%CI: 1.071–3.897), and the effect of gender on appreciable modification of light activities was significant (*P* = 0.024). The associations of other lifestyles, such as past drinker, vigorous-intensity activity, depression with multimorbidity were unchanged after stratification by gender, showing no statistically significant interactions.

**Table 4:** The association between lifestyles and multimorbidity among participants using global logistic model by gender.

Variable	gender		*P*-Interaction ^b^
	Male	female	
**Smoking (ref = Non-smoker)**			0.495
Current smoker	2.544(1.562,4.143) ^a^	1.637(0.478,5.601)	
Former smoker	2.514(1.509,4.189) ^a^	1.431(0.327,6.264)	
**Drinking (ref = Non-drinker)**			0.608
Current drinker	0.801(0.501,1.280)	1.081(0.678,1.724)	
Past drinker	3.146(1.817,5.445) ^a^	4.217(2.101,8.465) ^a^	
**Sleep Duration (ref = 6-8h)**			0.068
<6h	1.020(0.659,1.578)	0.948(0.715,1.257)	
>8h	1.137(0.556,2.329)	0.899(0.560,1.442)	
**Vigorous-Intensity Activity (ref = No)**			0.327
Yes	0.542(0.337,0.872) ^a^	0.648(0.471,0.891) ^a^	
**Moderate Activity (ref = No)**			0.773
Yes	0.933(0.616,1.412)	0.820(0.626,1.073)	
**Light Activities (ref = No)**			0.024
Yes	2.043(1.071,3.897) ^a^	0.951(0.681,1.327)	
**Depression (ref = No)**			0.090
Yes	1.931(1.287,2.898) ^a^	1.626(1.248,2.120) ^a^	

OR (95% CI) is given in the table.

^a^ represents a statistically significant OR value (P<0.05)

^b^ Statistically significant in interaction between gender and variables (P<0.05).

### Multivariate spatial logistic regression

This study also performed the GWLR analysis and determined the optimal bandwidth size was 7088.000. The local spatial effects of risk factors on multimorbidity were displayed in Table 5.

**Table 5:** The association between lifestyles and multimorbidity among participants using GWLR.

Variable	Mean^a^		Min^a^		Median^a^		Max^a^	
	β^b^	OR^c^	β^b^	OR^c^	β^b^	OR^c^	β^b^	OR^c^
**Intercept**	-3.141	0.043	-3.168	0.042	-3.145	0.043	-2.883	0.056
**Age (ref = 45–54)**								
55–64	0.189	1.208	0.140	1.150	0.189	1.208	0.368	1.445
65–74	0.422	1.525	0.386	1.471	0.422	1.525	0.467	1.595
≥75	0.244	1.276	0.224	1.251	0.244	1.276	0.310	1.363
**Gender (ref = Male)**								
Female	0.004	1.004	-0.048	0.953	0.004	1.004	0.030	1.030
**Smoking (ref = Non-drinker)**								
Current smoker	0.184	1.202	0.174	1.190	0.183	1.201	0.262	1.300
Former smoker	0.156	1.169	0.105	1.111	0.156	1.169	0.225	1.252
**Drinking (ref = Non-drinker)**								
Current drinker	-0.023	0.977	-0.053	0.948	-0.024	0.976	0.095	1.100
Former drinker	0.211	1.235	0.117	1.124	0.212	1.236	0.215	1.240
**Sleep Duration (ref = 6-8h)**								
<6h	-0.005	0.995	-0.008	0.992	-0.006	0.994	0.015	1.015
>8h	-0.015	0.985	-0.019	0.981	-0.016	0.984	0.068	1.070
**Vigorous-Intensity Activity (ref = No)**								
Yes	-0.220	0.803	-0.401	0.670	-0.219	0.803	-0.190	0.827
**Moderate Activity (ref = No)**								
Yes	-0.078	0.925	-0.090	0.914	-0.079	0.924	-0.025	0.975
**Light Activities (ref = No)**								
Yes	0.053	1.054	-0.017	0.983	0.054	1.055	0.071	1.074
**Depression (ref = No)**								
Yes	0.258	1.294	0.236	1.266	0.258	1.294	0.367	1.443

^a^ Mean, Min, Median and Max: Mean, minimum, medium, and maximum denote the minimum, median, and maximum local estimate values, respectively

^b^ β is the estimated effect of the independent variable

^c^ OR: odds ratio is adjusted OR.

**Fig 2:**
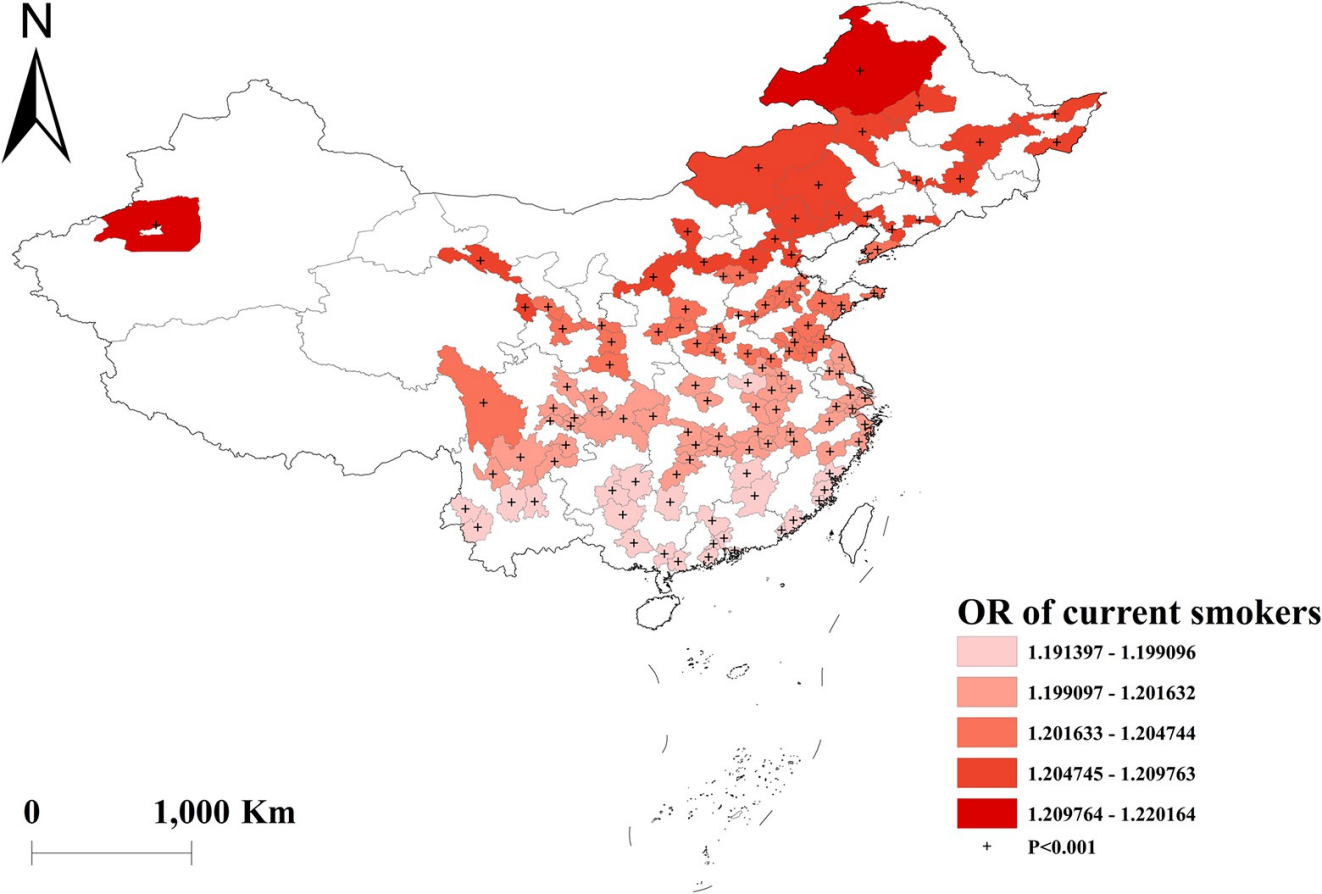
Geographical distribution of the adjusted ORs for current smokers in GWLR model.

**Fig 3:**
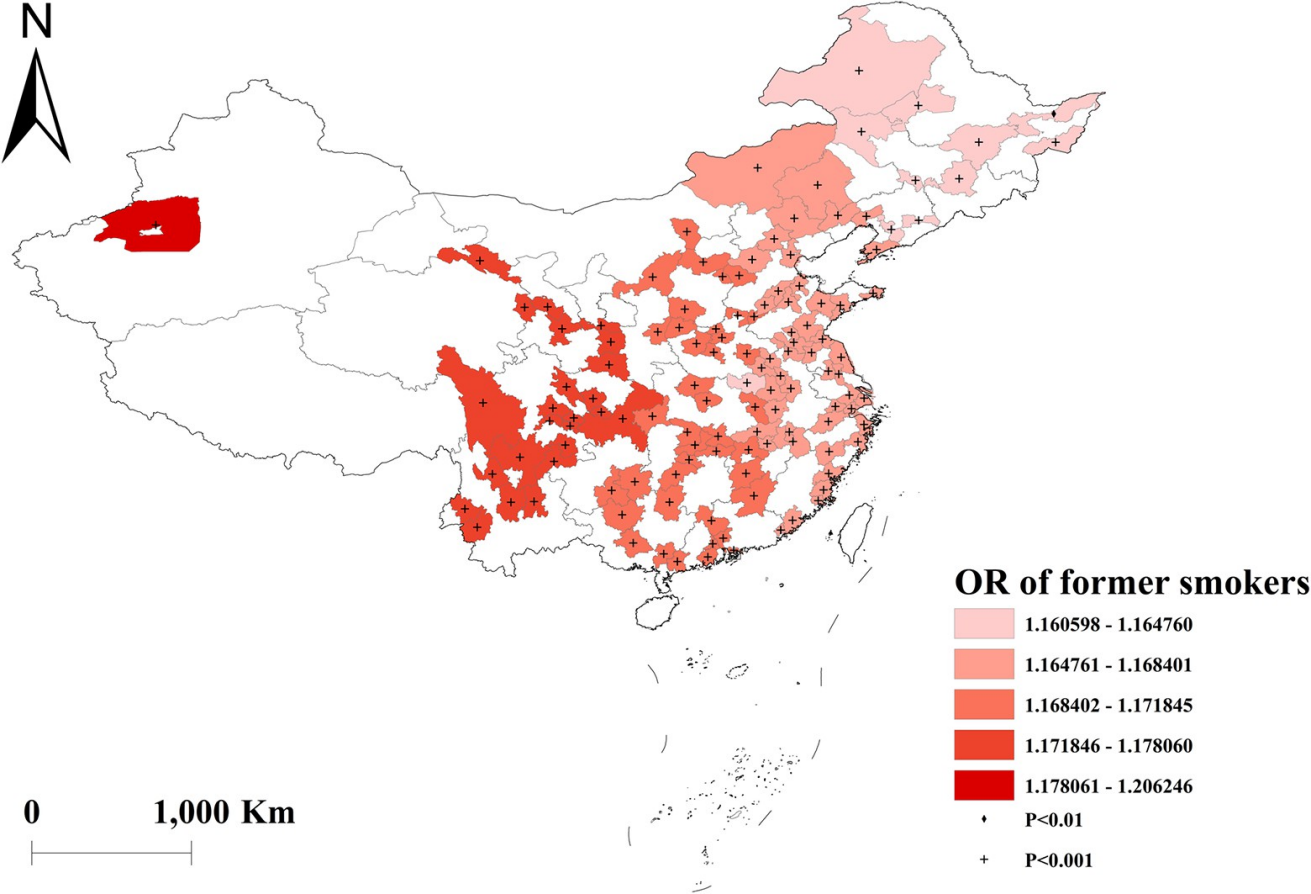
Geographical distribution of the adjusted ORs for former smokers in GWLR model.

**Fig 4:**
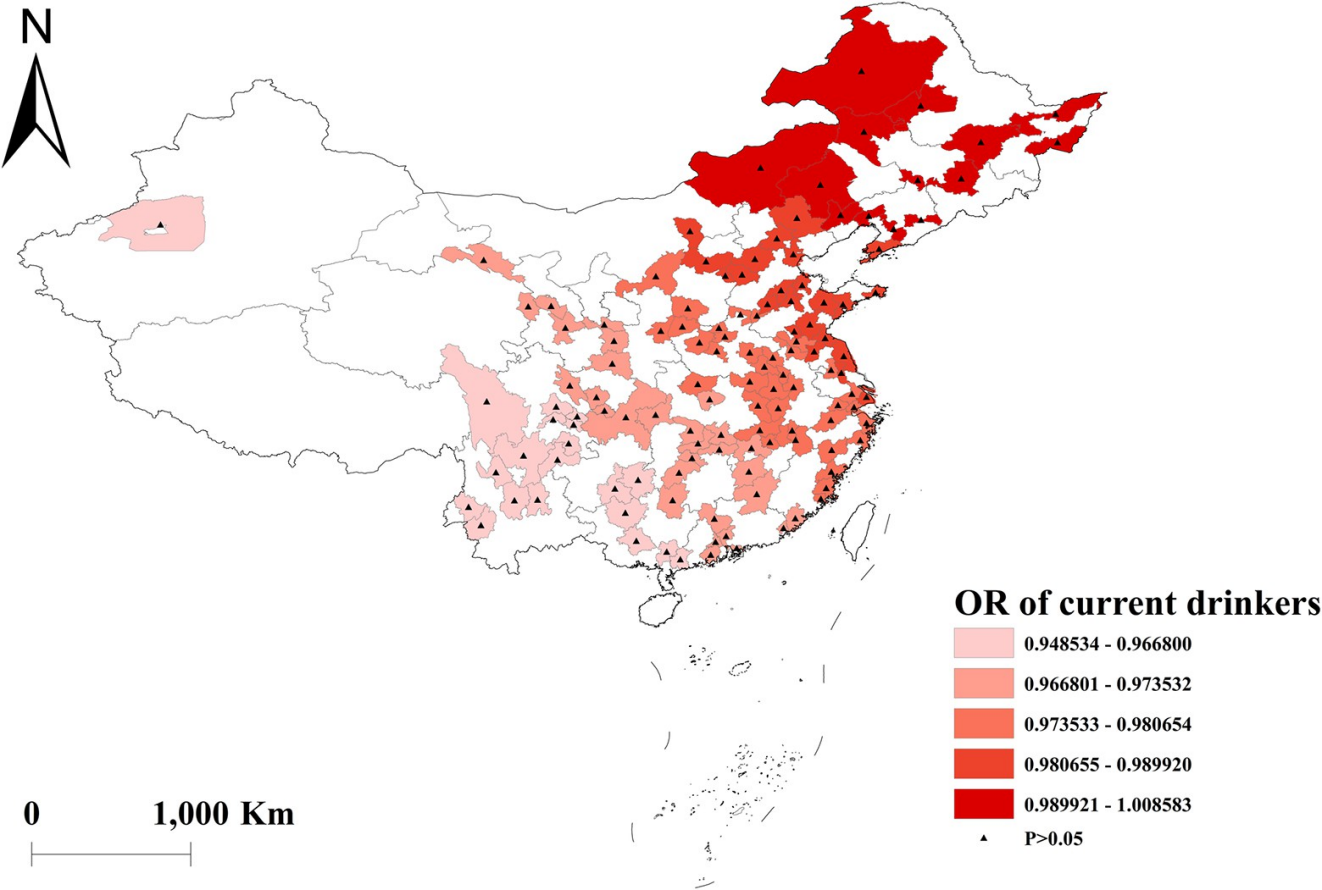
Geographical distribution of the adjusted ORs for current drinkers in GWLR model.

**Fig 5:**
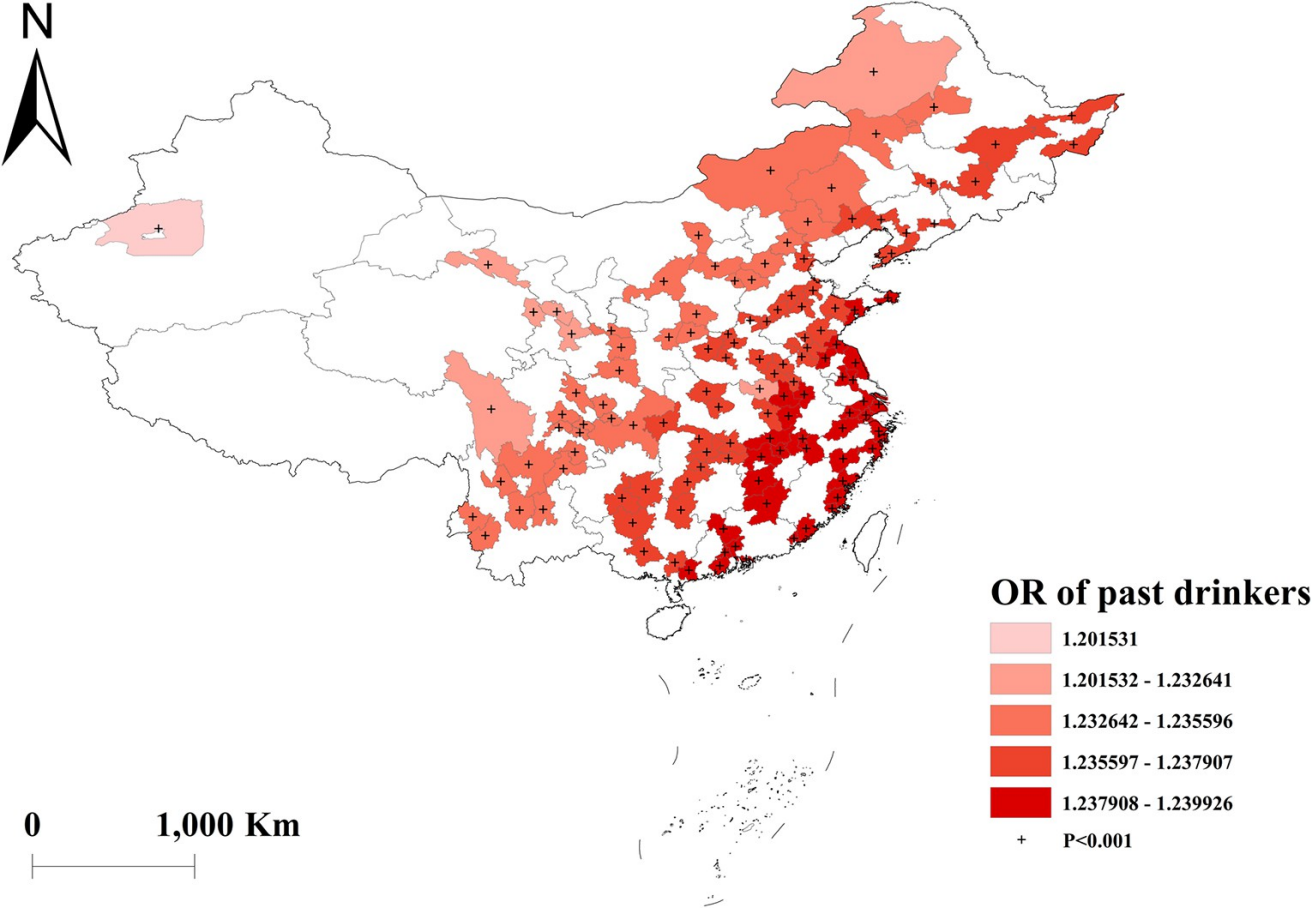
Geographical distribution of the adjusted ORs for past drinkers in GWLR model.

**Fig 6:**
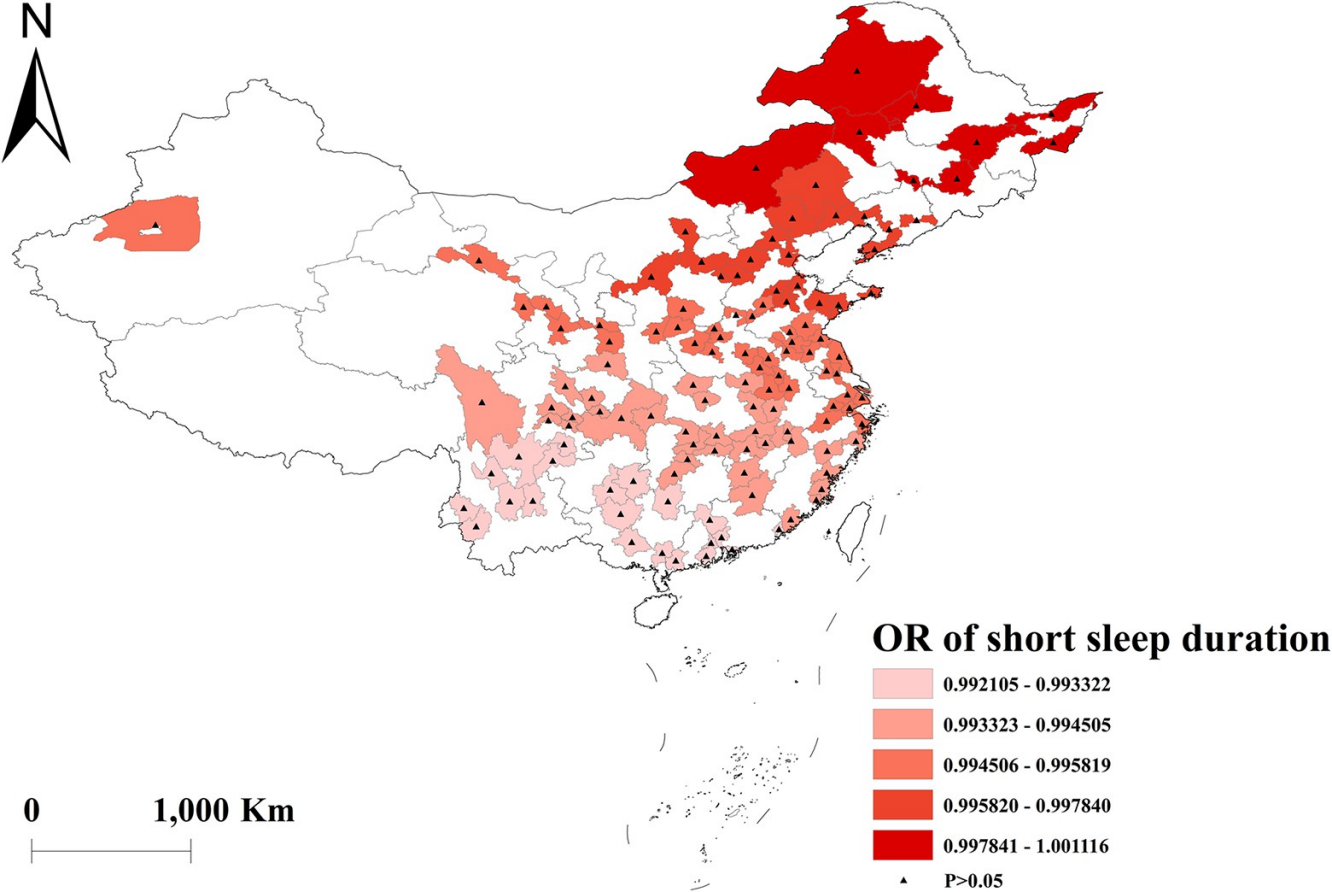
Geographical distribution of the adjusted ORs for short sleep duration in GWLR model.

**Fig 7:**
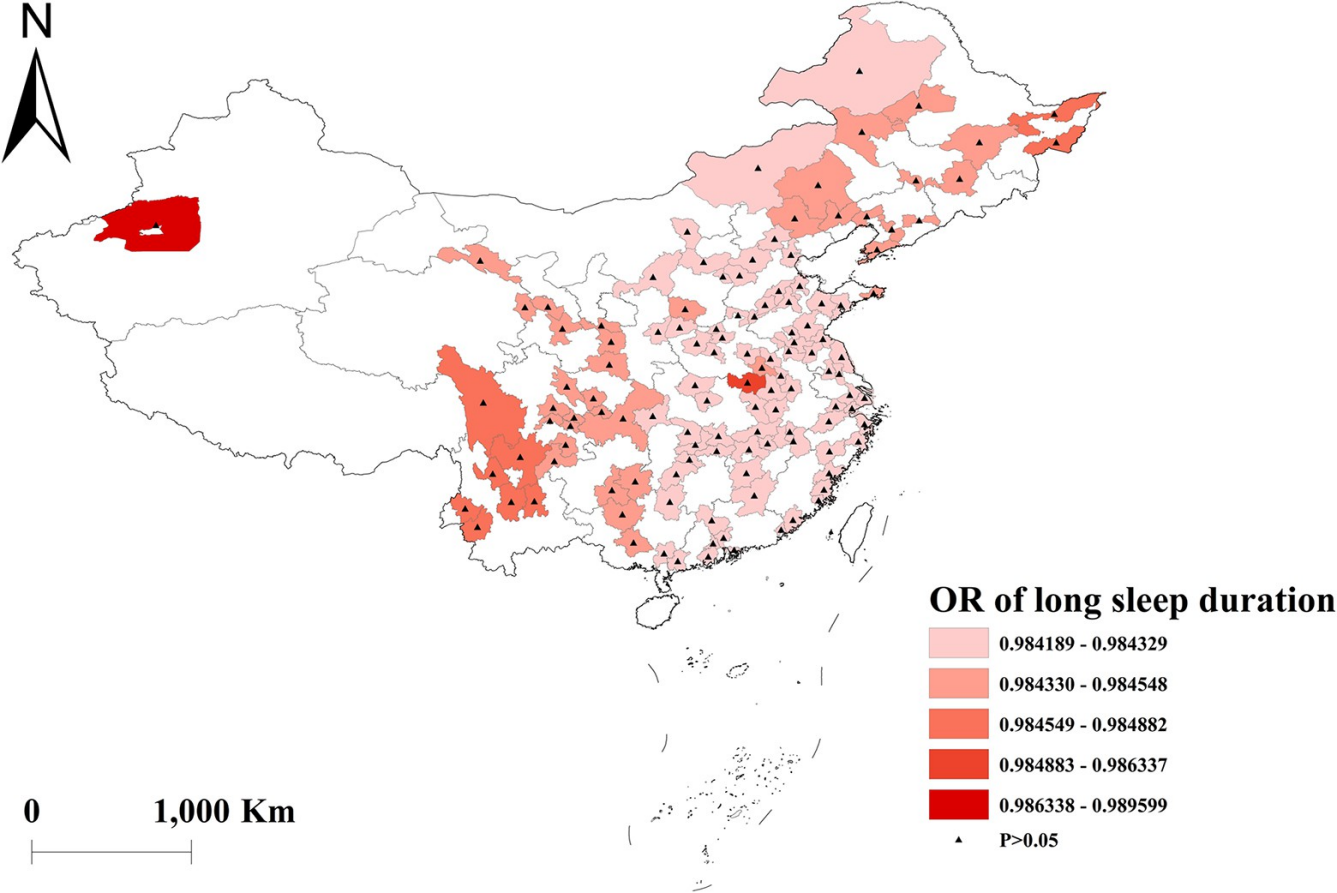
Geographical distribution of the adjusted ORs for long sleep duration in GWLR model.

The GWLR analysis indicated that current smokers and former smokers were more likely to develop multimorbidity. The highest OR values for current smokers were observed in north, whereas the lowest OR values were in south (Fig 2). For former smokers, the highest OR was in southwest, and the lowest OR was in northeast (Fig 3). However, current drinkers did not show a significant influence on multimorbidity (Fig 4). Past drinkers were an important risk factor for developing multimorbidity, with the highest OR in eastern China and the lowest value in the northwest China (Fig 5). Nonetheless, there was no clear association between sleep duration and multimorbidity (Figs 6 and 7).

**Fig 8:**
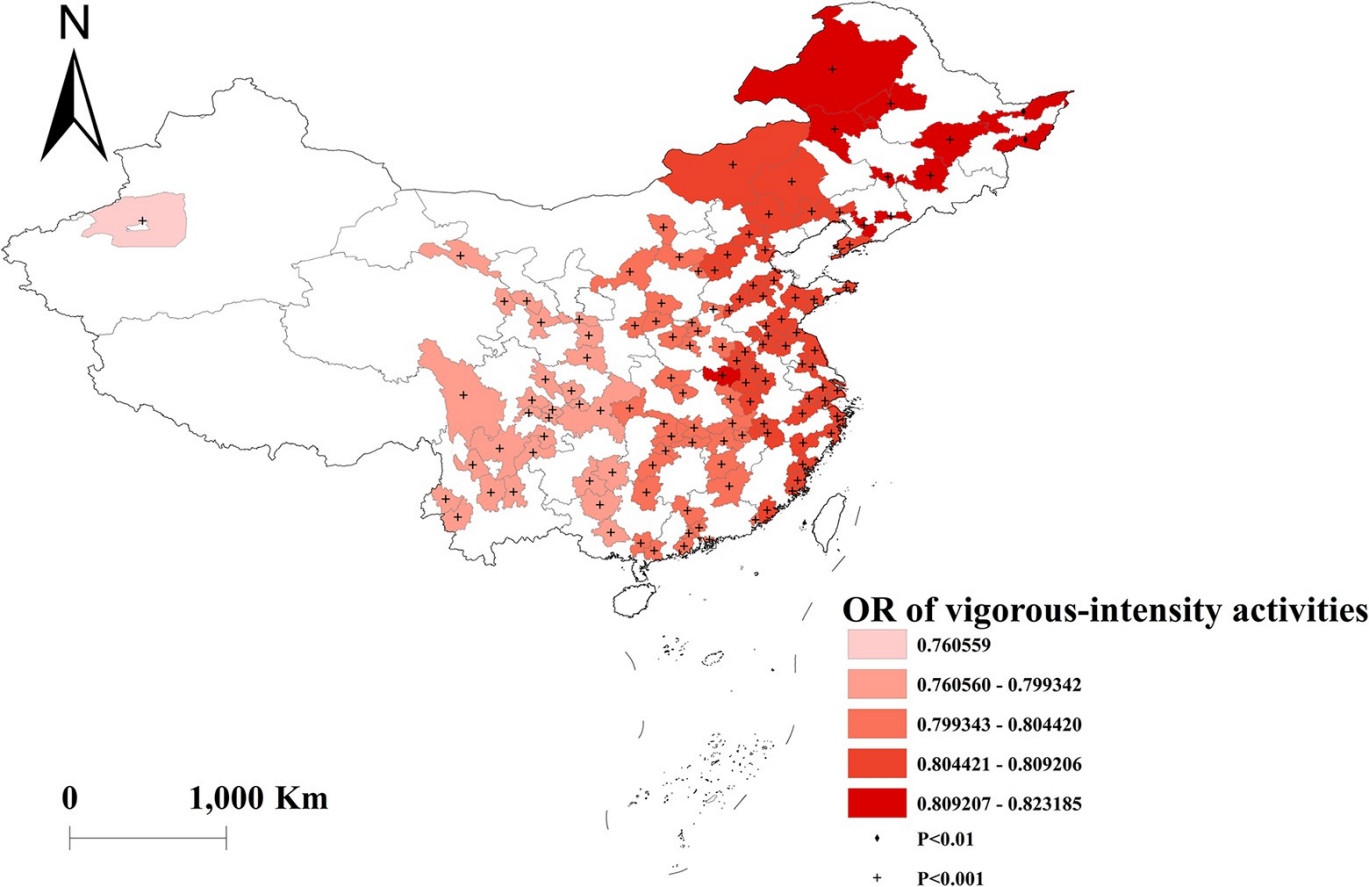
Geographical distribution of the adjusted ORs for vigorous-intensity activities in GWLR model.

**Fig 9:**
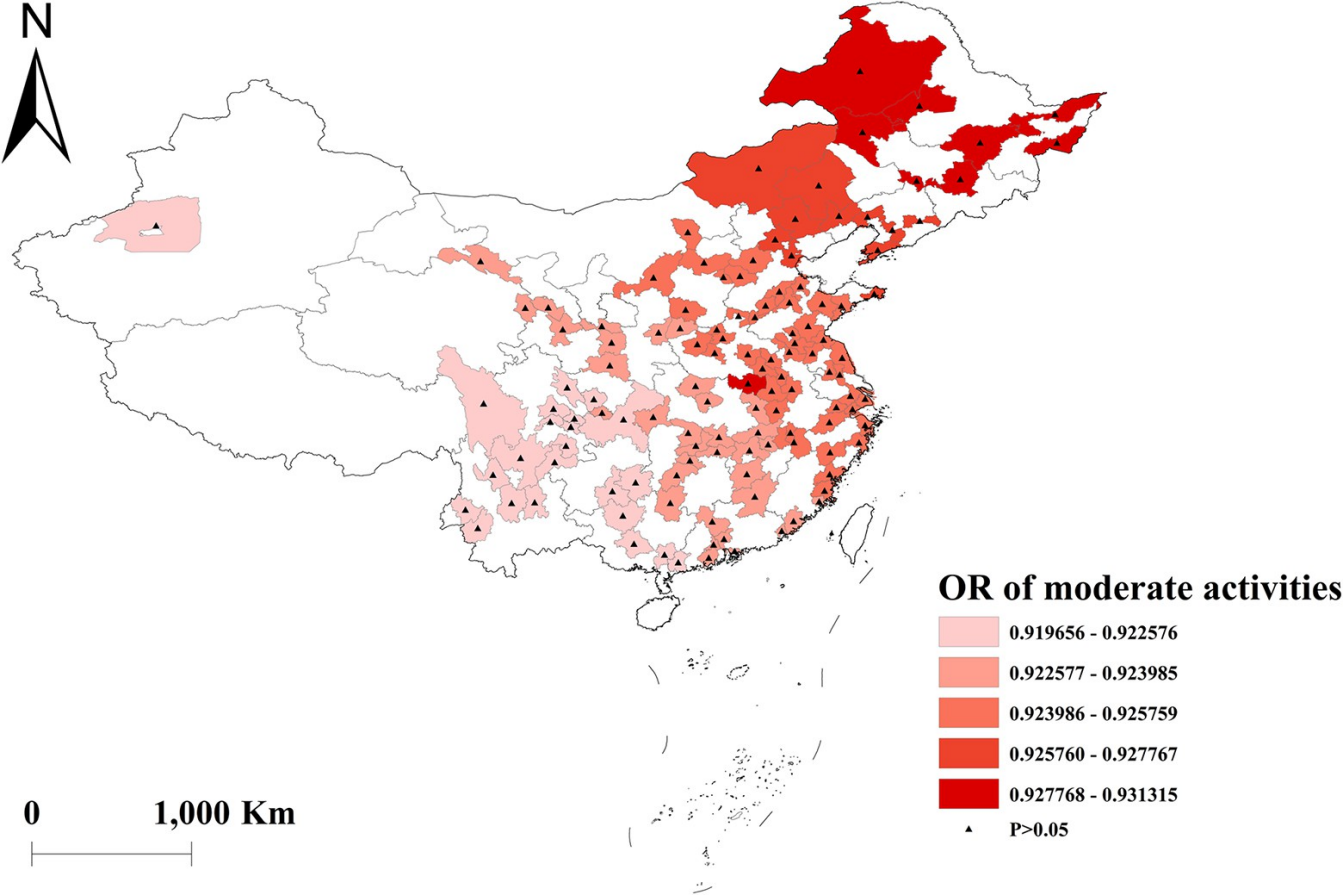
Geographical distribution of the adjusted ORs for moderate activities in GWLR model.

**Fig 10:**
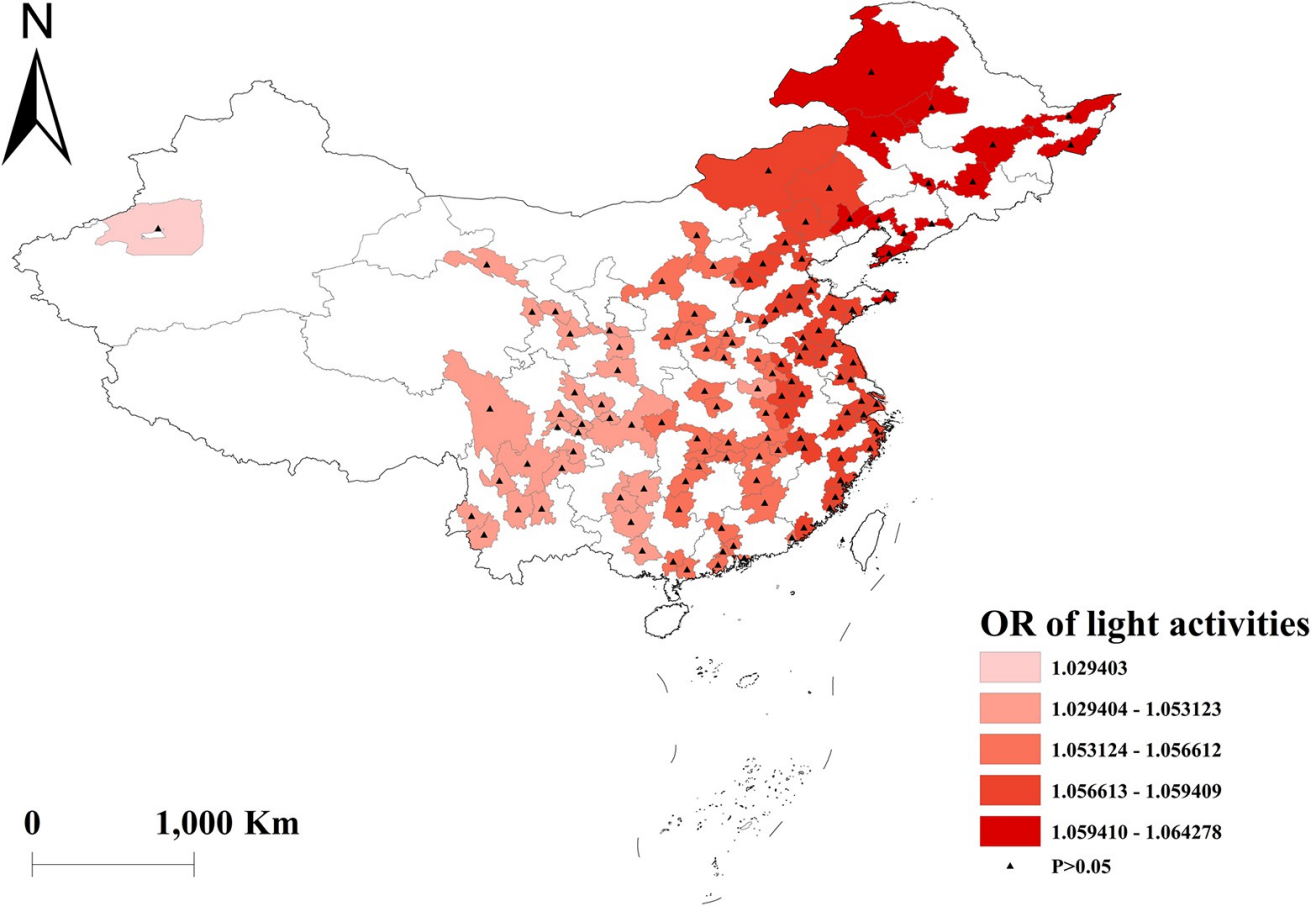
Geographical distribution of the adjusted ORs for light activities in GWLR model.

**Fig 11:**
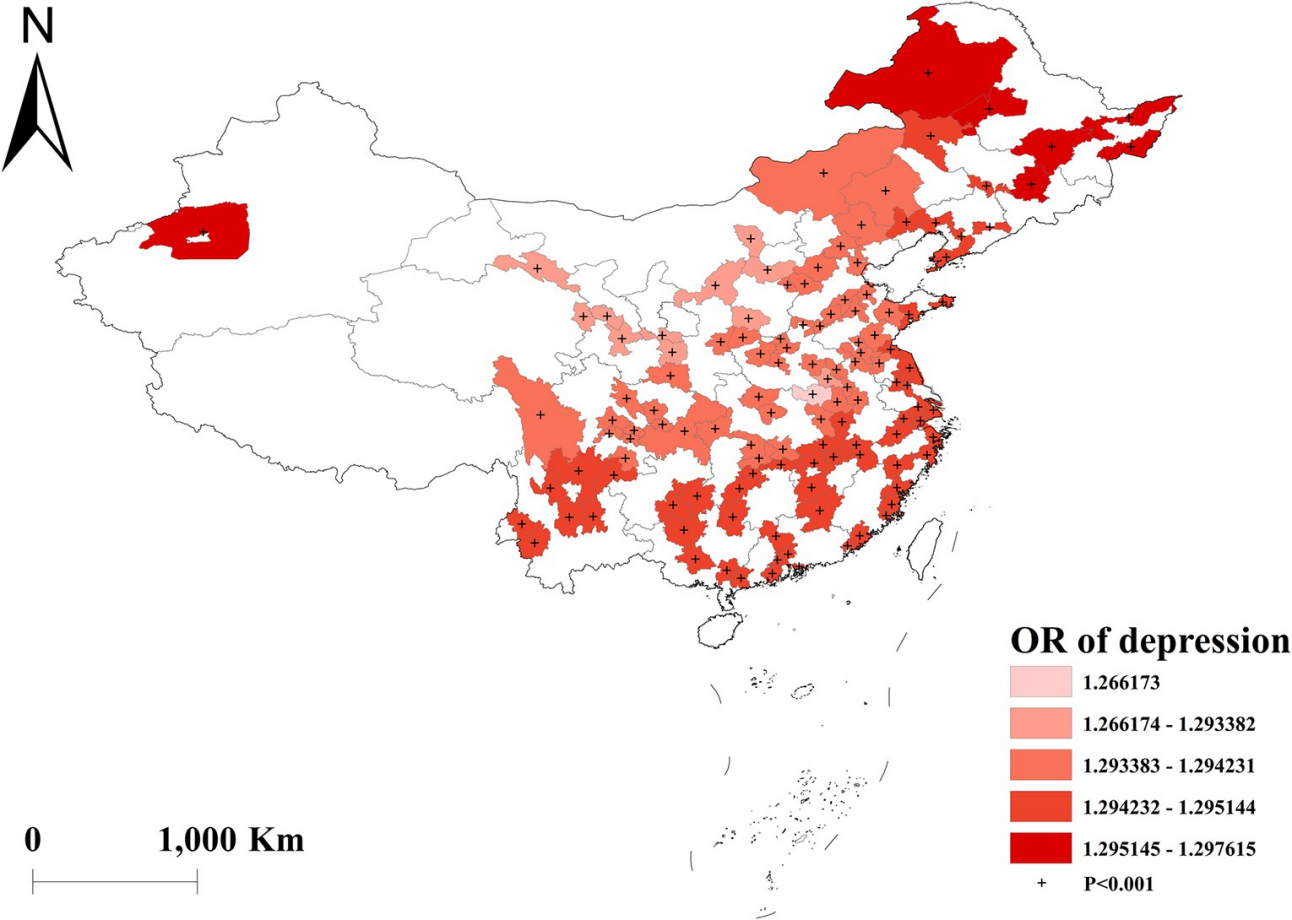
Geographical distribution of the adjusted ORs for depression in GWLR model.

Vigorous-intensity activities were negatively correlated with multimorbidity, with the highest protective effect in western China (Fig 8). And there was no effect of moderate and light activities on multimorbidity across the country (Figs 9 and 10). Depressed participants in central China were less likely to acquire multimorbidity (Fig 11).

The gender-specific relationship between lifestyles and multimorbidity is similar to our main results using the GWLR model (Tables 6 and 7). The relationship between current and former smokers and multimorbidity was consistent with our main findings, but only among male and not female (S1, S2, S11 and S12 Figs). Current drinkers were positively associated with multimorbidity among men only in west (S3 Fig), while past drinkers may increase the risk of multimorbidity among women, particularly in the south (S14 Fig). In men, light activities increased the risk of multimorbidity only in east (S9 Fig). The relationship between sleep duration or depression and multimorbidity when stratified by gender was consisted with our main results.

**Table 6:** The association between lifestyles and multimorbidity among participants using GWLR among male.

Variable	Mean^a^		Min^a^		Median^a^		Max^a^	
	β^b^	OR^c^	β^b^	OR^c^	β^b^	OR^c^	β^b^	OR^c^
**Intercept**	-4.199	0.015	-4.469	0.011	-4.238	0.014	-2.585	0.075
**Age (ref = 45–54)**								
55–64	0.293	1.340	0.106	1.112	0.299	1.349	0.694	2.002
65–74	0.631	1.879	0.236	1.266	0.629	1.876	0.813	2.255
≥75	0.949	2.583	0.511	1.667	0.927	2.527	1.660	5.259
**Smoking (ref = Non-drinker)**								
Current smoker	0.990	2.691	0.367	1.443	0.991	2.694	1.370	3.935
Former smoker	0.873	2.394	0.259	1.296	0.879	2.408	1.399	4.051
**Drinking (ref = Non-drinker)**								
Current drinker	-0.329	0.720	-0.799	0.450	-0.300	0.741	0.218	1.244
Former drinker	1.215	3.370	0.557	1.745	1.240	3.456	1.325	3.762
**Sleep Duration (ref = 6-8h)**								
<6h	0.104	1.110	-0.183	0.833	0.111	1.117	0.155	1.168
>8h	0.147	1.158	-0.440	0.644	0.161	1.175	0.878	2.406
**Vigorous-Intensity Activity (ref = No)**								
Yes	-0.499	0.607	-1.162	0.313	-0.476	0.621	-0.099	0.906
**Moderate Activity (ref = No)**								
Yes	-0.050	0.951	-0.196	0.822	-0.046	0.955	0.181	1.198
**Light Activities (ref = No)**								
Yes	0.756	2.130	0.218	1.244	0.767	2.153	0.828	2.289
**Depression (ref = No)**								
Yes	0.584	1.793	0.221	1.247	0.583	1.791	1.054	2.869

^a^ Mean, Min, Median and Max: Mean, minimum, medium, and maximum denote the minimum, median, and maximum local estimate values, respectively

^b^ β is the estimated efect of the independent variable

^c^ OR: odds ratio is adjusted OR.

**Table 7:** The association between lifestyles and multimorbidity among participants using GWLR among female.

Variable	Mean^a^		Min^a^		Median^a^		Max^a^	
	β^b^	OR^c^	β^b^	OR^c^	β^b^	OR^c^	β^b^	OR^c^
**Intercept**	-3.676	0.025	-3.912	0.020	-3.655	0.026	-3.326	0.036
**Age (ref = 45–54)**								
55–64	0.530	1.699	0.233	1.262	0.543	1.721	0.871	2.389
65–74	1.288	3.626	1.222	3.394	1.287	3.622	1.369	3.931
≥75	0.981	2.667	0.648	1.912	0.977	2.656	1.213	3.364
**Smoking (ref = Non-drinker)**								
Current smoker	-0.432	0.649	-2.940	0.053	-0.337	0.714	1.012	2.751
Former smoker	0.448	1.565	-0.462	0.630	0.553	1.738	1.085	2.959
**Drinking (ref = Non-drinker)**								
Current drinker	-0.001	0.999	-0.397	0.672	-0.050	0.951	0.471	1.602
Former drinker	1.347	3.846	0.953	2.593	1.327	3.770	1.685	5.392
**Sleep Duration (ref = 6-8h)**								
<6h	-0.075	0.928	-0.147	0.863	-0.070	0.932	0.077	1.080
>8h	-0.147	0.863	-0.274	0.760	-0.152	0.859	0.004	1.004
**Vigorous-Intensity Activity (ref = No)**								
Yes	-0.285	0.752	-0.733	0.480	-0.262	0.770	-0.105	0.900
**Moderate Activity (ref = No)**								
Yes	-0.217	0.805	-0.301	0.740	-0.220	0.803	-0.068	0.934
**Light Activities (ref = No)**								
Yes	-0.011	0.989	-0.315	0.730	0.017	1.017	0.198	1.219
**Depression (ref = No)**								
Yes	0.458	1.581	0.413	1.511	0.452	1.571	0.651	1.917

^a^ Mean, Min, Median and Max: Mean, minimum, medium, and maximum denote the minimum, median, and maximum local estimate values, respectively

^b^ β is the estimated efect of the independent variable

^c^ OR: odds ratio is adjusted OR.

## Discussion

In this study, we aimed to investigate the geographic distribution of lifestyles and multimorbidity across different regions in China’s adults. We employed the GWLR model to explore the regional differences in the relationship between lifestyles and multimorbidity. Our findings demonstrate that the prevalence of multimorbidity in adults varied among surveyed areas, and the association between lifestyles and multimorbidity showed regional variation.

Our study reported a total prevalence of approximately 5.13% of multimorbidity. Among those with multimorbidity, the prevalence of hypertension was highest (86.81%), followed by heart disease (59.07%), diabetes or high blood sugar (45.33%) and stroke (27.47%). The prevalence of multimorbidity varied widely among different studies, depending on participants’ age and the number of chronic diseases included [17]. Gu et al. reported a multimorbidity rate of about 56.5% in the elderly over 60 years old, with hypertension having the highest prevalence (48.2%) among thirteen chronic conditions [18]. A cross-sectional survey in LMICs, including China, reported that the prevalence of multimorbidity containing eight chronic diseases was highest in Russia (34.7%) and lowest in China (20.3%) [19]. Some studies in China have also presented the prevalence of multimorbidity with two or more chronic conditions ranging from 5% to over 50% [18, 20–22]. Since our study only included four chronic diseases, the prevalence of multimorbidity was lower than that in other studies but was consistent with some studies in China.

Compared to non-smokers, former and current smokers were found to be more likely to suffer from multimorbidity using global regression and GWLR model. Studies have proven that tobacco contains various toxic and carcinogenic compounds that can lead to various diseases, including cardiovascular, cancer, respiratory, and neurological diseases [23]. For example, in a population-based biomedical cohort study in Australia, current smokers were 1.71 times more likely to develop multimorbidity than non-smokers [24]. Using the GWLR model, our study revealed that current smokers had highest risk of multimorbidity in northern China, while former smokers was most likely to suffer from multimorbidity in western China. Moreover, a review by Pan et al. indicated that smoking has a greater effect on males than females, which is consistent with our study findings [25]. In the surveyed regions of northern and western China, most participants were from rural areas, and the levels of economic development was lower than in other regions of China. Previous studies have shown that individuals with a low socioeconomic status are more likely to be smokers, and smoking is more prevalent in rural areas of western and northeastern China [26, 27]. These results underscore the importance of controlling tobacco smoking among the elderly, particularly in northern and western regions among males. However, additional studies are needed to corroborate these geographical variations.

Previous studies provides evidence of a causal relationship between alcohol consumption and increased risk of stroke and cardiovascular disease [28, 29]. Our study found that past drinkers were more likely to develop multimorbidity compared to current drinkers, according to both global logistic regression and GWLR models. However, we observed significant geographic variations that reflect China’s geographic, economic, and cultural diversity. Specifically, the highest risk for multimorbidity among past drinkers was in eastern China, while the lowest was in the northwest. Gender was also linked to alcohol consumption, with men preferring alcohol because men who drank seen as masculine and faithful [30]. In the areas of alcohol production, mainly located in eastern China, such as Anhui, Jiangsu, and Shandong, the proportion of alcohol consumption was relatively higher compared to the national level [31]. Moreover, in eastern China, there are more migrant workers from less economically developed areas, which may have led to poorer psychological conditions and unhealthy lifestyles, including excessive alcohol consumption [32, 33]. These reasons may partly explain the regional differences in the association between past drinkers and multimorbidity.

According to the results of the GWLR, engaging in vigorous-intensity activities was associated with a decreased risk of multimorbidity, and this effect was more pronounced in western China. Li et al. reported that physical inactivity was responsible for 6–10% of non-communicable disease deaths worldwide, and the proportion was even higher for certain diseases (e.g., 30% for ischemic heart disease) [34]. Physical activity has been shown to improve cardiovascular and respiratory fitness, as well as bone and functional health in adults [35]. In our study, the proportion of physical activity was generally higher in western China compared to eastern China. This could be due to heavier workloads and less leisure time available for exercise in the latter region, where there is greater employment and work competition. These might in part contribute to the geographic-specific pattern in the relationship between vigorous-intensity activities and multimorbidity.

The global logistic regression found that depression may bring about a higher risk of multimorbidity in the population, which was consistent with the findings of the Australian Work Outcomes Research Cost-benefit study and the 2013–2014 Canadian Community Health Survey [36, 37]. Depression and multimorbidity are known to co-occur in elderly individuals, leading to accelerated aging [38]. The GWLR model further revealed regional differences, with the association between depression and multimorbidity being weakest in central China and no gender difference. This could be attributed to the stable economy and lower psychosocial stress levels in the region [39]. However, further evidence is needed to determine geographical variations in the inconsistent results reported for the associations between depression and multimorbidity.

This study has several strengths, including the wide coverage across China with a sample from 28 provinces that can be generalized to the entire country. In addition, the use of the GWLR model to examine local variations provided maps for visualizing differences in multimorbidity-related lifestyles of adults between regions. However, the study has limitations that should be considered. First, the cross-sectional design of the study limits the ability to establish temporal and causal relationships between explanatory variables and multimorbidity. Second, the diagnoses of hypertension, diabetes or high blood sugar, heart disease, and stroke were based on self-reported information, which may have resulted in underestimation of the true prevalence of multimorbidity. Despite these limitations, our study is the first to explore the geographic variation in the association of lifestyles with multimorbidity among adults in China from a spatial perspective, and fills in some gaps in current research.

## Conclusions

In summary, smoking is a significant risk factor for multimorbidity in male adults, with current and former smokers at higher risk in north and west regions, respectively. However, no correlation was found between smoking and multimorbidity among female. Past drinkers were associated with increased risk of multimorbidity, especially in eastern China, for men but not for women. Vigorous-intensity activities were found to significantly decrease the risk of disease in west and no gender difference. Light activities increased the risk of multimorbidity among males only in east China. Depression appear to increase the risk for multimorbidity, with the weakest effects in central China and no gender difference. These findings provide valuable clues for tailoring site-specific intervention strategies based on geographical variations.

## Supporting information

S1 Fig Geographical distribution of the adjusted ORs for current smokers in GWLR model among male. (TIF)

S2 Fig Geographical distribution of the adjusted ORs for past smokers in GWLR model among male. (TIF)

S3 Fig Geographical distribution of the adjusted ORs for current drinkers in GWLR model among male. (TIF)

S4 Fig Geographical distribution of the adjusted ORs for past drinkers in GWLR model among male. (TIF)

S5 Fig Geographical distribution of the adjusted ORs for short sleep duration in GWLR model among male. (TIF)

S6 Fig Geographical distribution of the adjusted ORs for long sleep duration in GWLR model among male. (TIF)

S7 Fig Geographical distribution of the adjusted ORs for vigorous-intensity activities in GWLR model among male. (TIF)

S8 Fig Geographical distribution of the adjusted ORs for moderate activities in GWLR model among male. (TIF)

S9 Fig Geographical distribution of the adjusted ORs for light activities in GWLR model among male. (TIF)

S10 Fig Geographical distribution of the adjusted ORs for depression in GWLR model among male. (TIF)

S11 Fig Geographical distribution of the adjusted ORs for current smokers in GWLR model among female. (TIF)

S12 Fig Geographical distribution of the adjusted ORs for past smokers in GWLR model among female. (TIF)

S13 Fig Geographical distribution of the adjusted ORs for current drinkers in GWLR model among female. (TIF)

S14 Fig Geographical distribution of the adjusted ORs for past drinkers in GWLR model among female. (TIF)

S15 Fig Geographical distribution of the adjusted ORs for short sleep duration in GWLR model among female. (TIF)

S16 Fig Geographical distribution of the adjusted ORs for long sleep duration in GWLR model among female. (TIF)

S17 Fig Geographical distribution of the adjusted ORs for vigorous-intensity activities in GWLR model among female. (TIF)

S18 Fig Geographical distribution of the adjusted ORs for moderate activities in GWLR model among female. (TIF)

S19 Fig Geographical distribution of the adjusted ORs for light activities in GWLR model among female. (TIF)

S20 Fig Geographical distribution of the adjusted ORs for depression in GWLR model among female. (TIF)

S1 File (DOCX)
