# Supplementary figures and images for: Geographical specific association between lifestyles and multimorbidity among adults in China
Source: PLoS One. 2023 Jun 7;18(6):e0286401. doi: 10.1371/journal.pone.0286401 (PMC10246811; doi:10.1371/journal.pone.0286401)

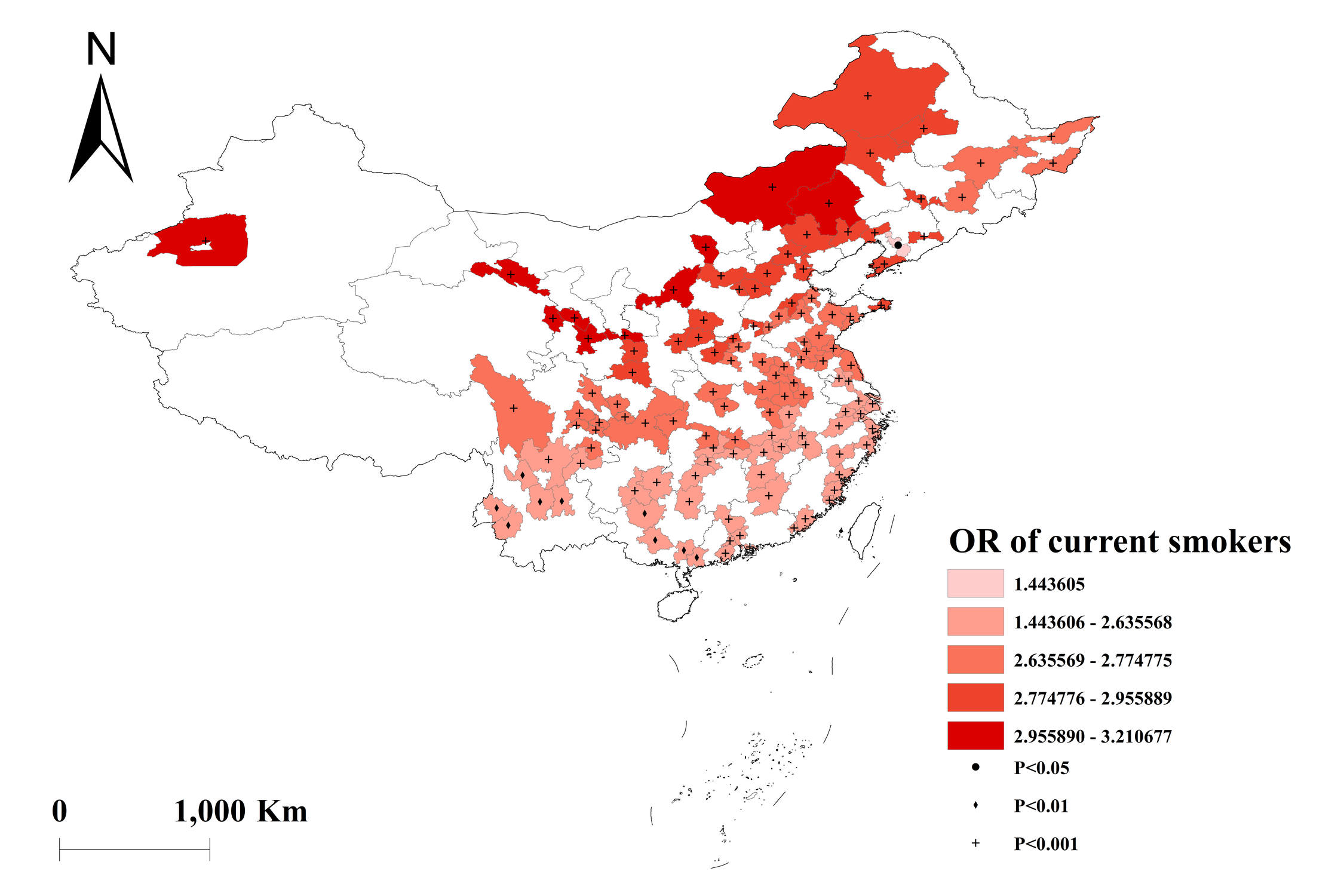

Supplement: S1 Fig — (TIF) [file pone.0286401.s001.tif]

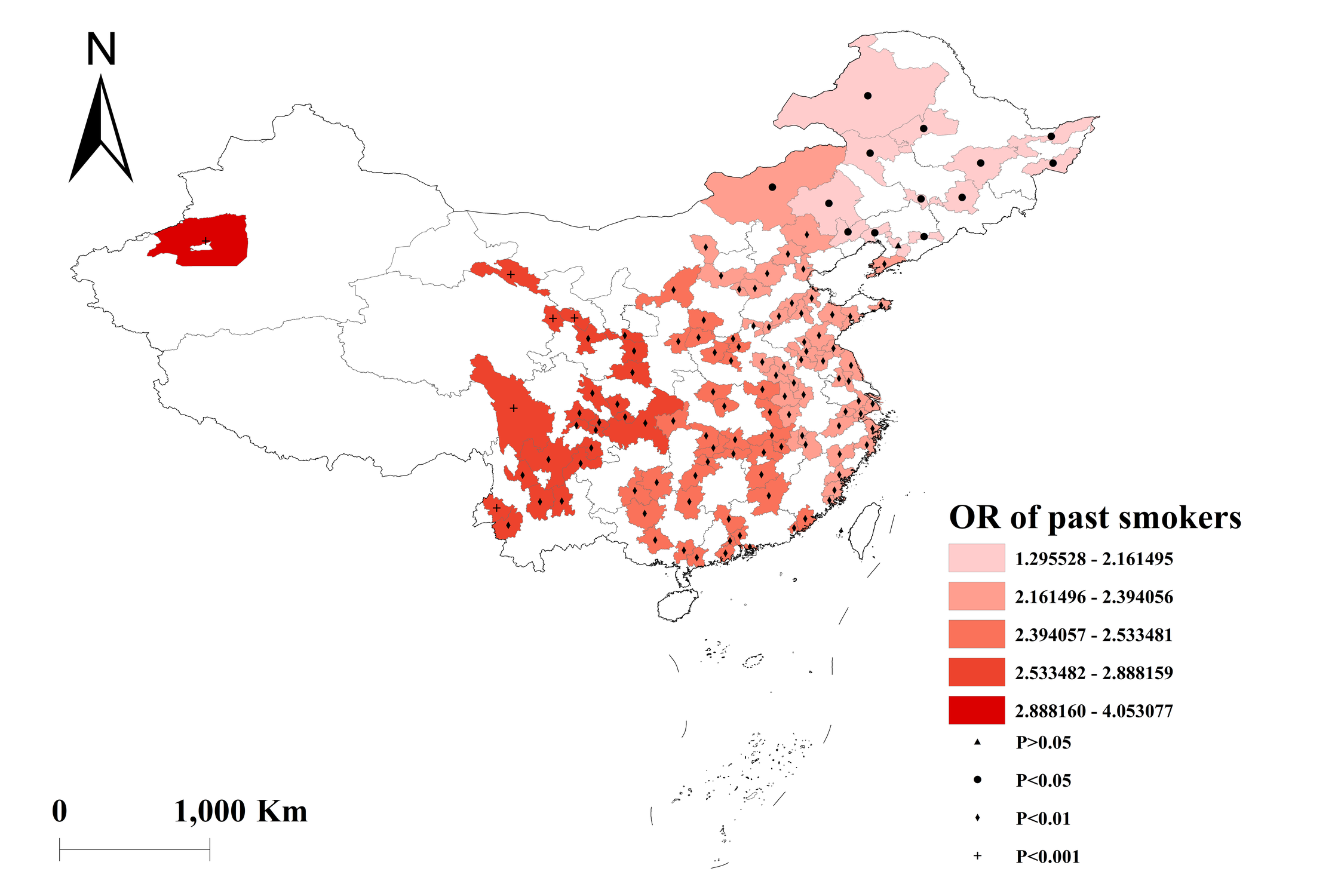

Supplement: S2 Fig — (TIF) [file pone.0286401.s002.tif]

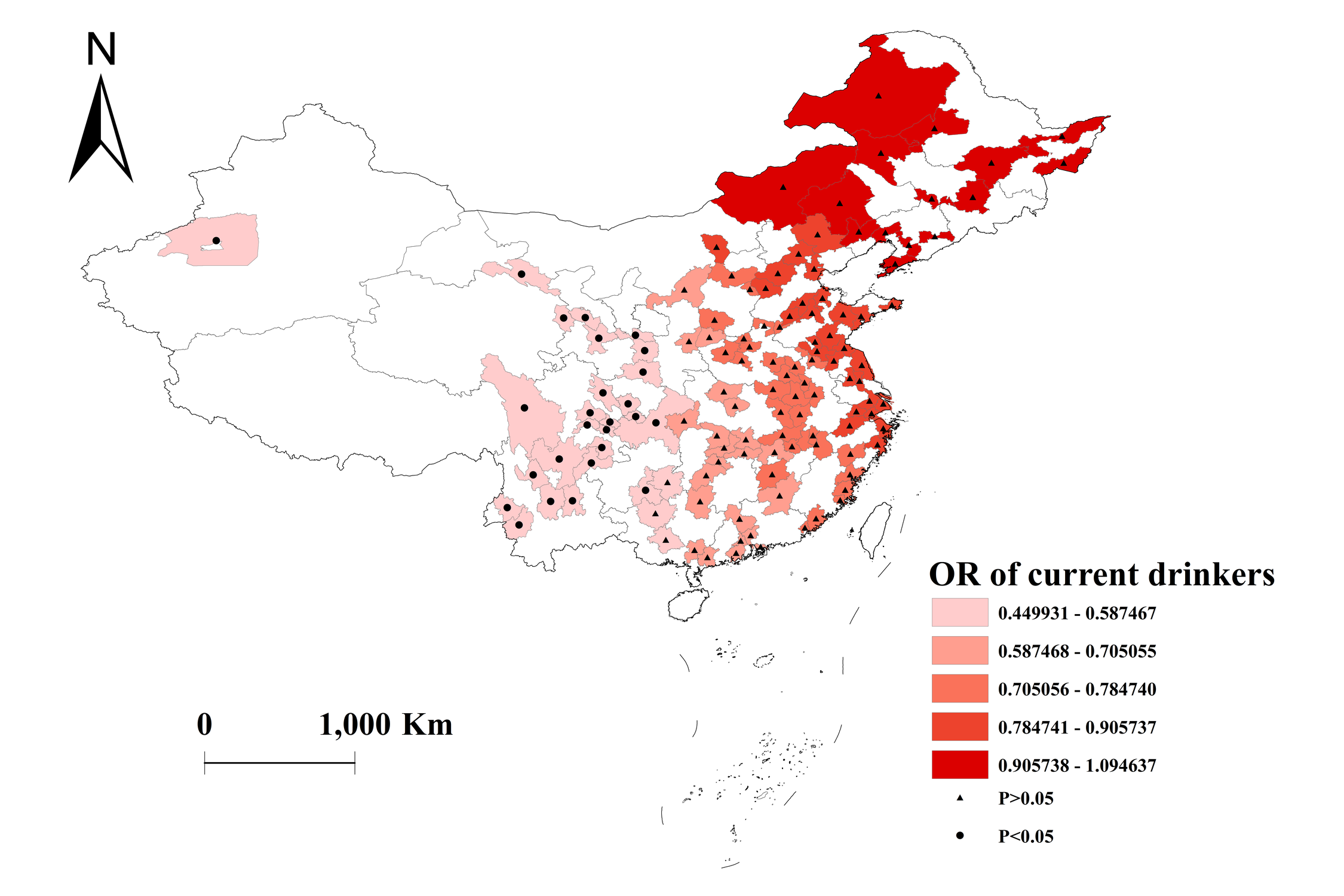

Supplement: S3 Fig — (TIF) [file pone.0286401.s003.tif]

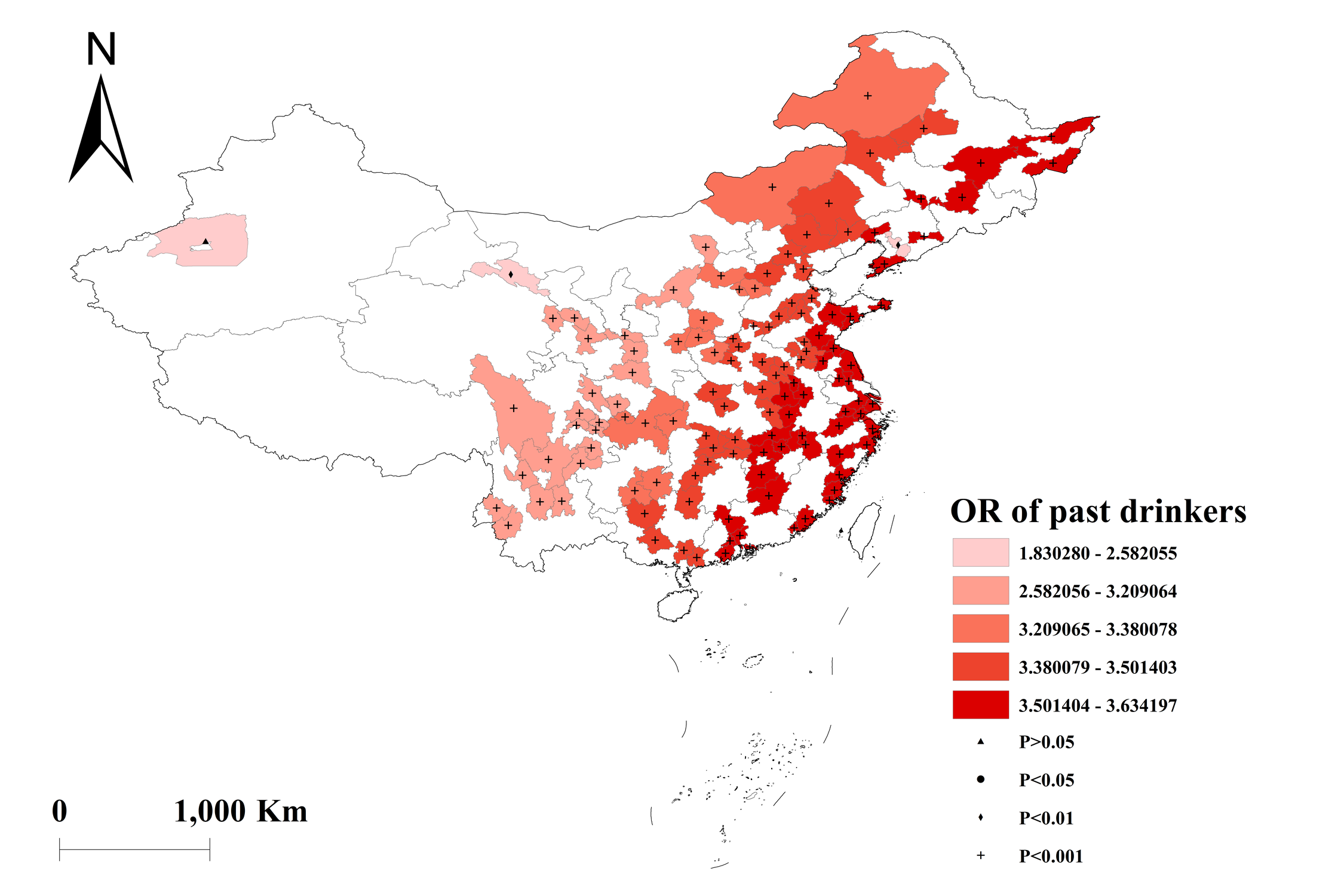

Supplement: S4 Fig — (TIF) [file pone.0286401.s004.tif]

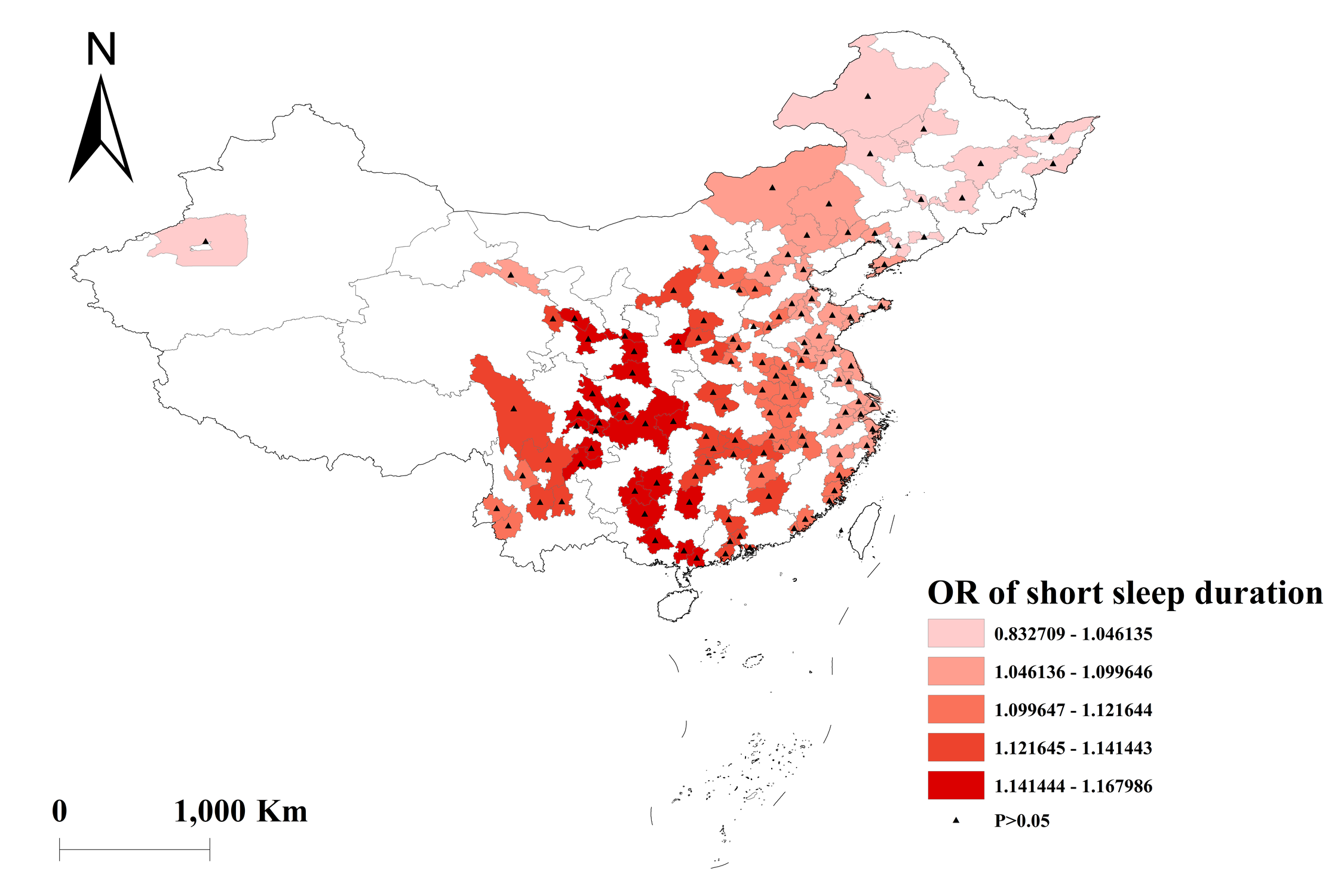

Supplement: S5 Fig — (TIF) [file pone.0286401.s005.tif]

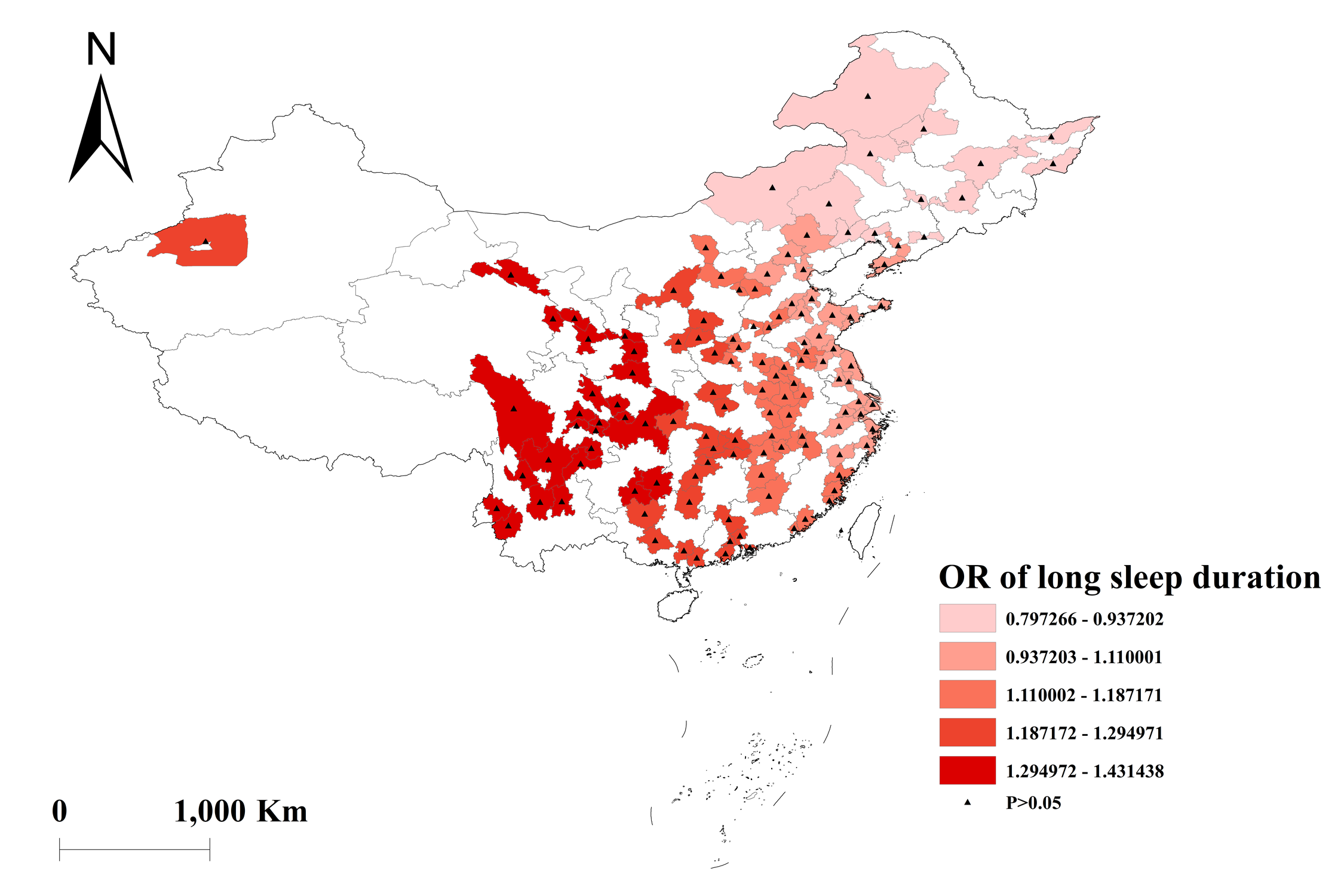

Supplement: S6 Fig — (TIF) [file pone.0286401.s006.tif]

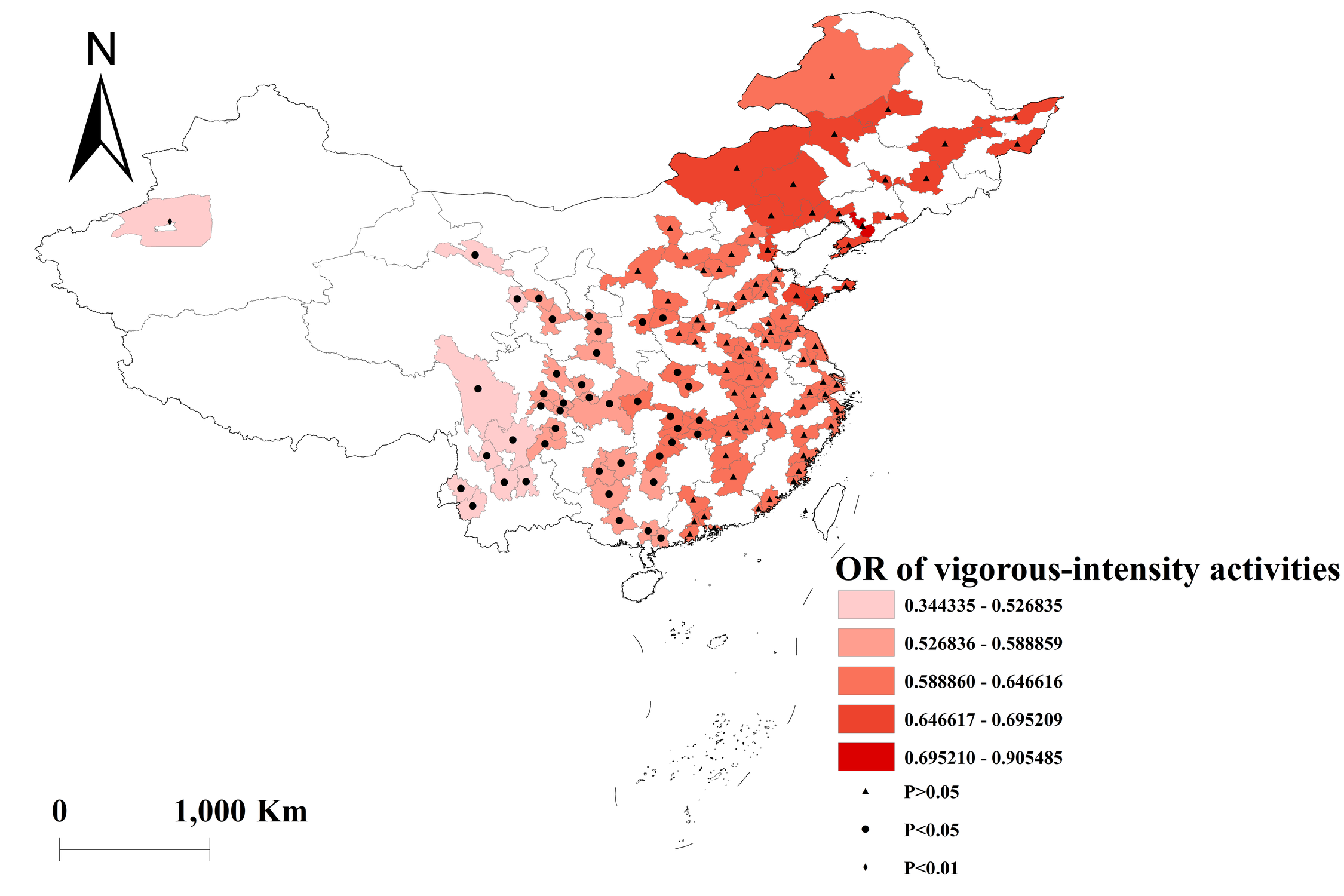

Supplement: S7 Fig — (TIF) [file pone.0286401.s007.tif]

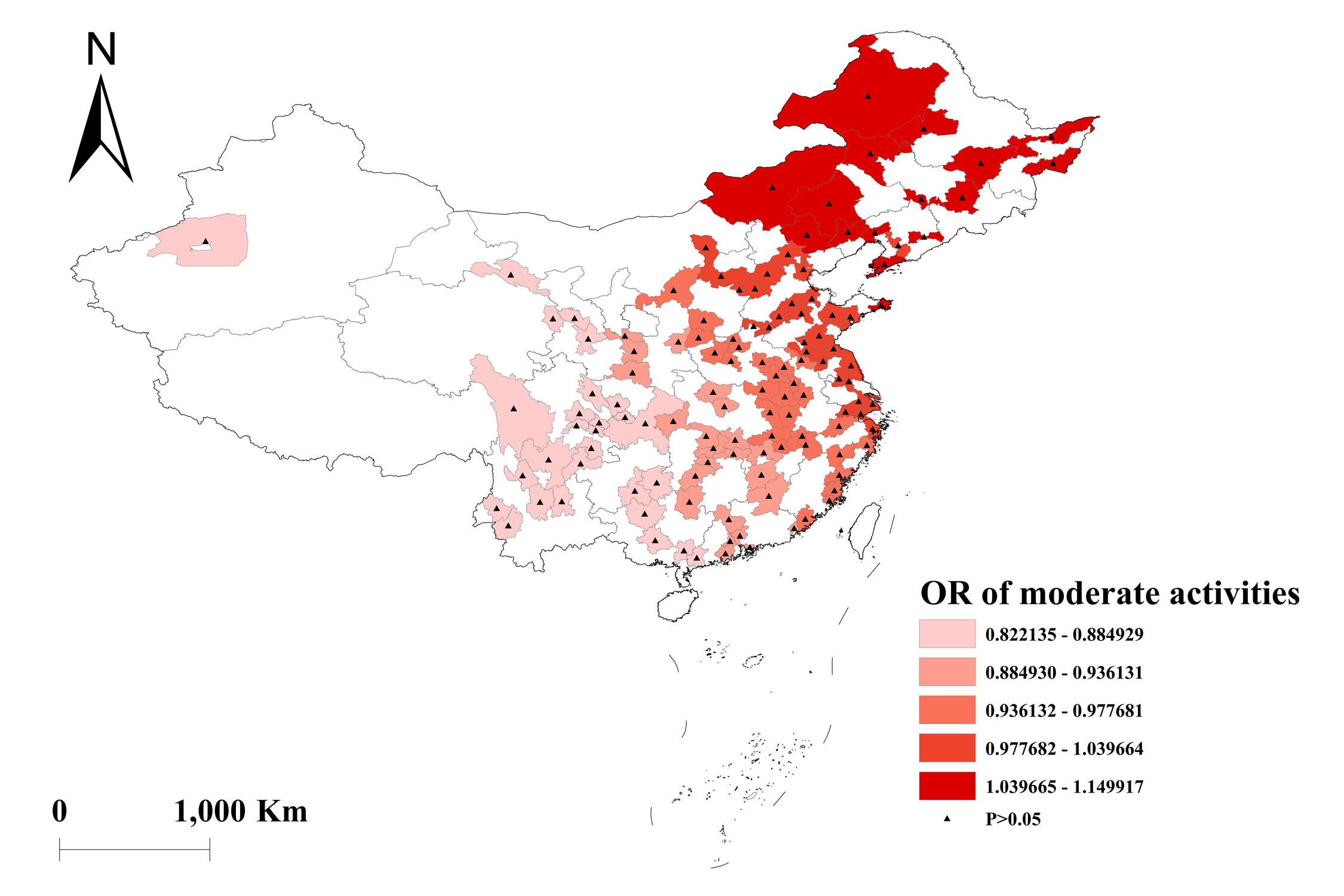

Supplement: S8 Fig — (TIF) [file pone.0286401.s008.tif]

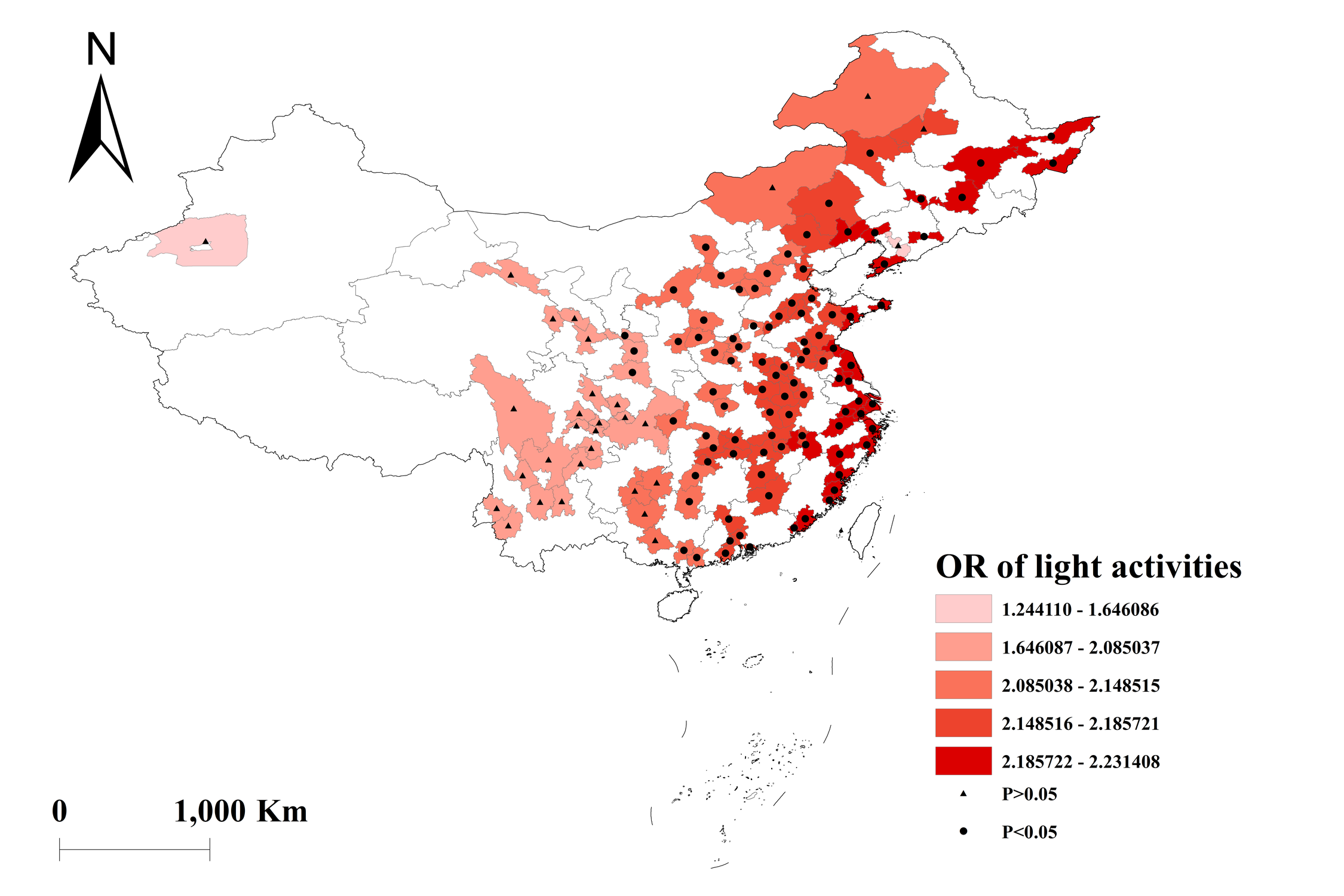

Supplement: S9 Fig — (TIF) [file pone.0286401.s009.tif]

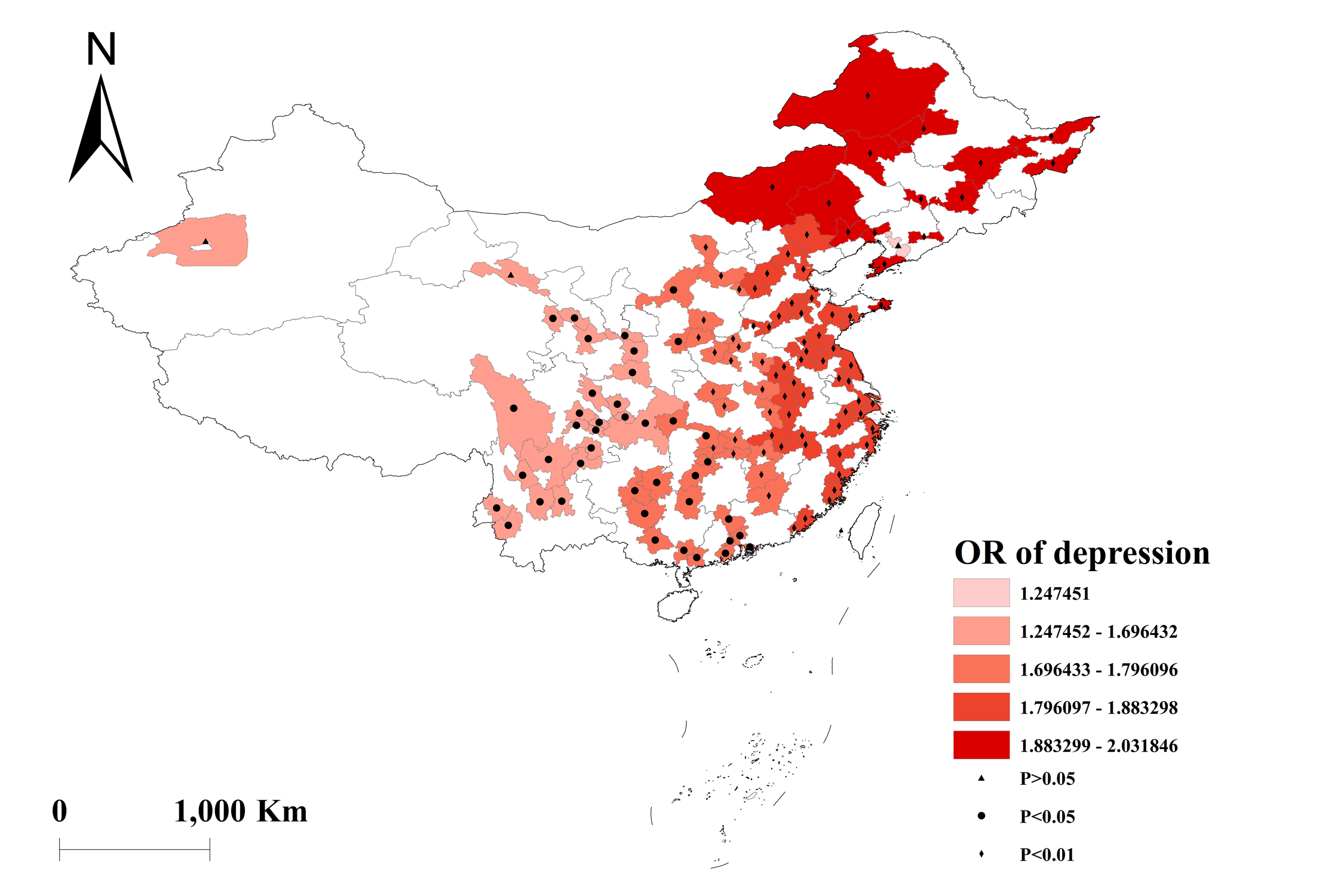

Supplement: S10 Fig — (TIF) [file pone.0286401.s010.tif]

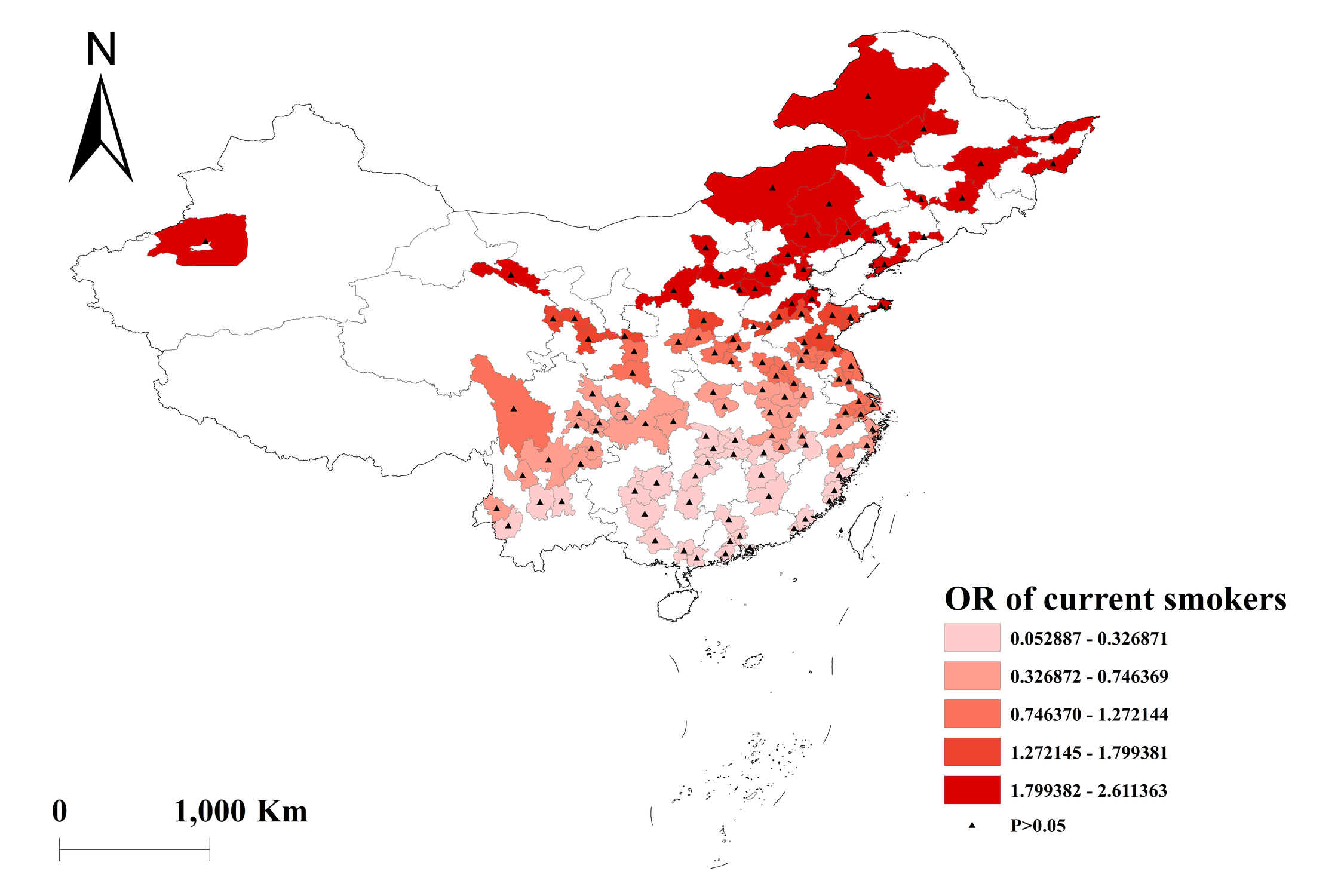

Supplement: S11 Fig — (TIF) [file pone.0286401.s011.tif]

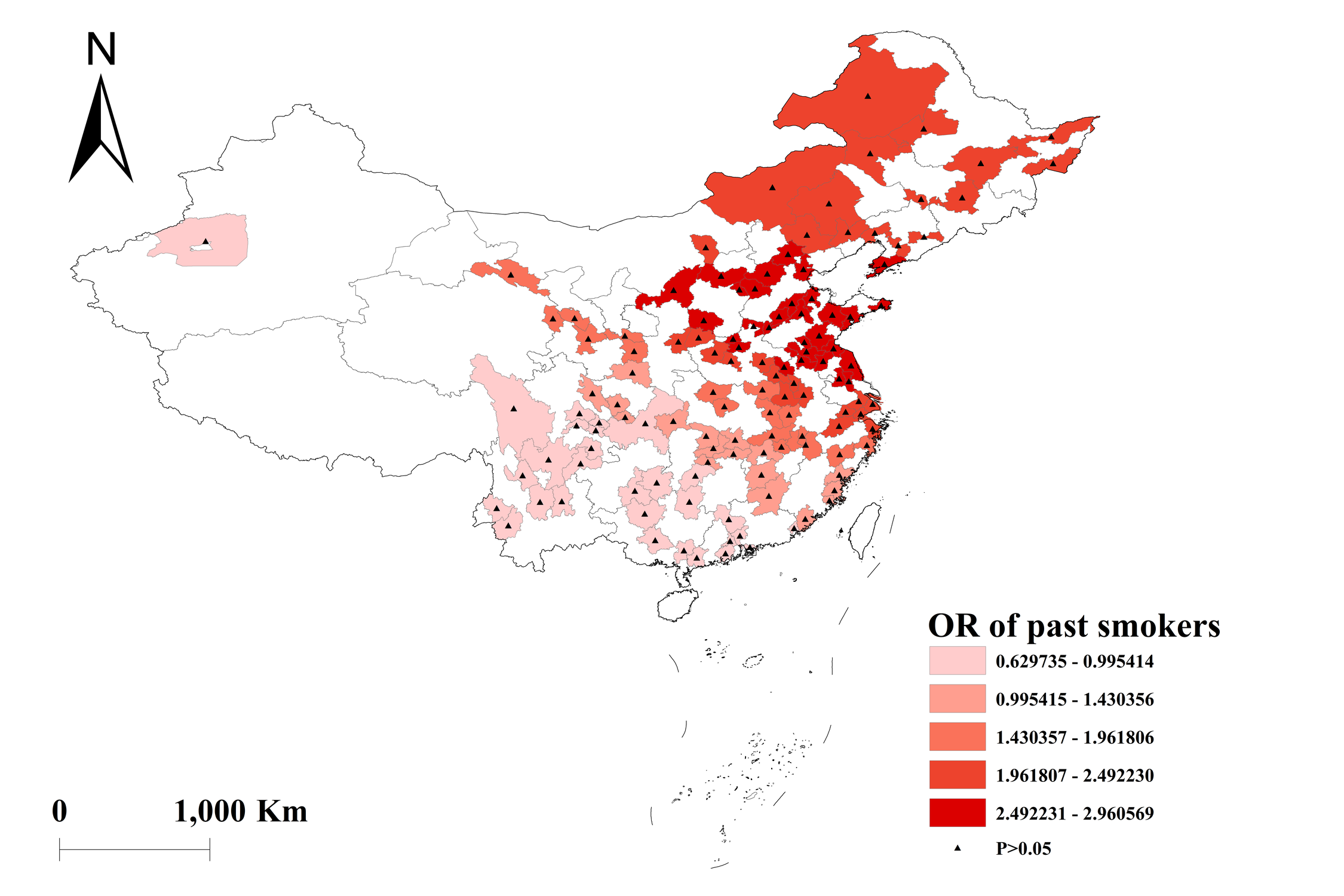

Supplement: S12 Fig — (TIF) [file pone.0286401.s012.tif]

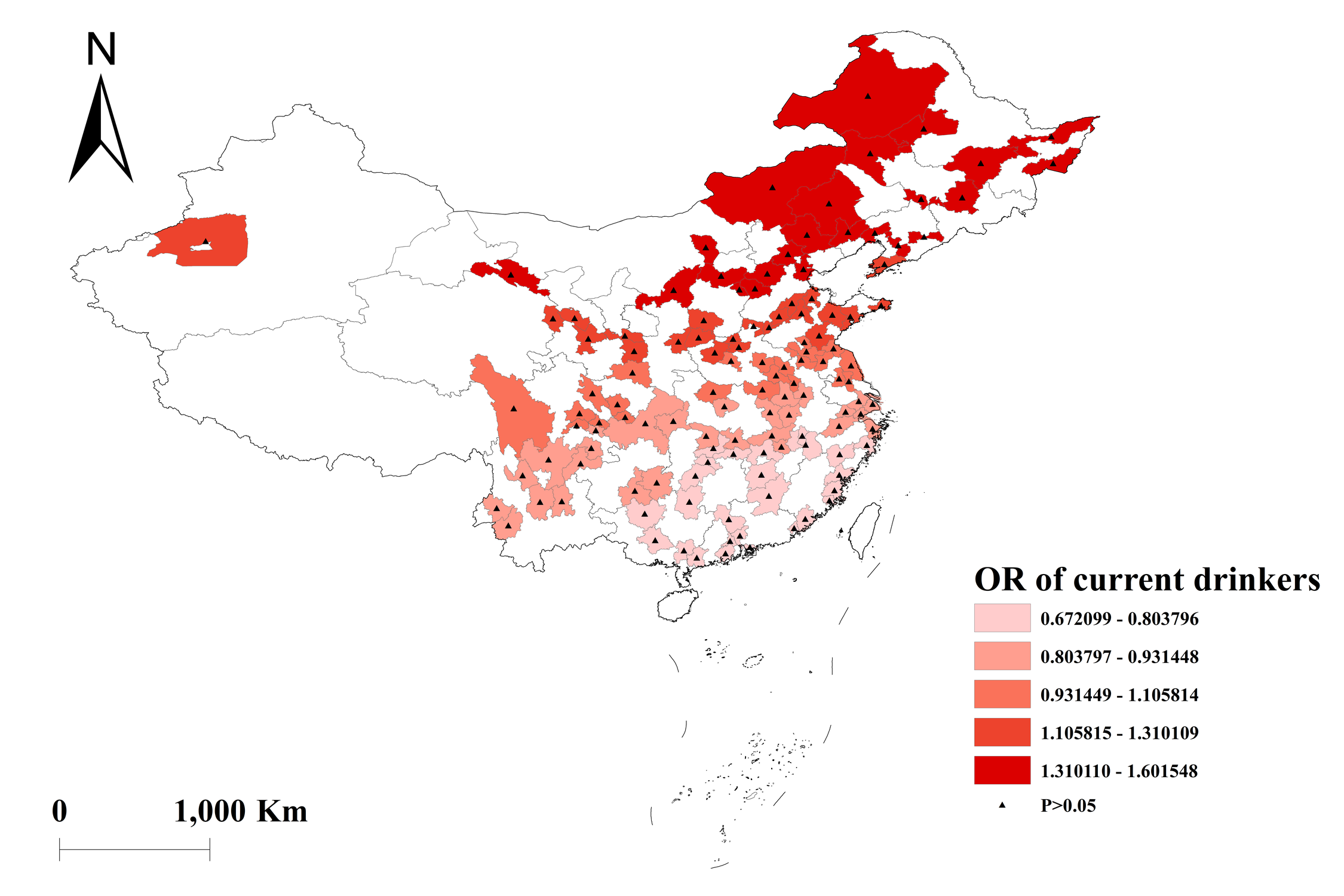

Supplement: S13 Fig — (TIF) [file pone.0286401.s013.tif]

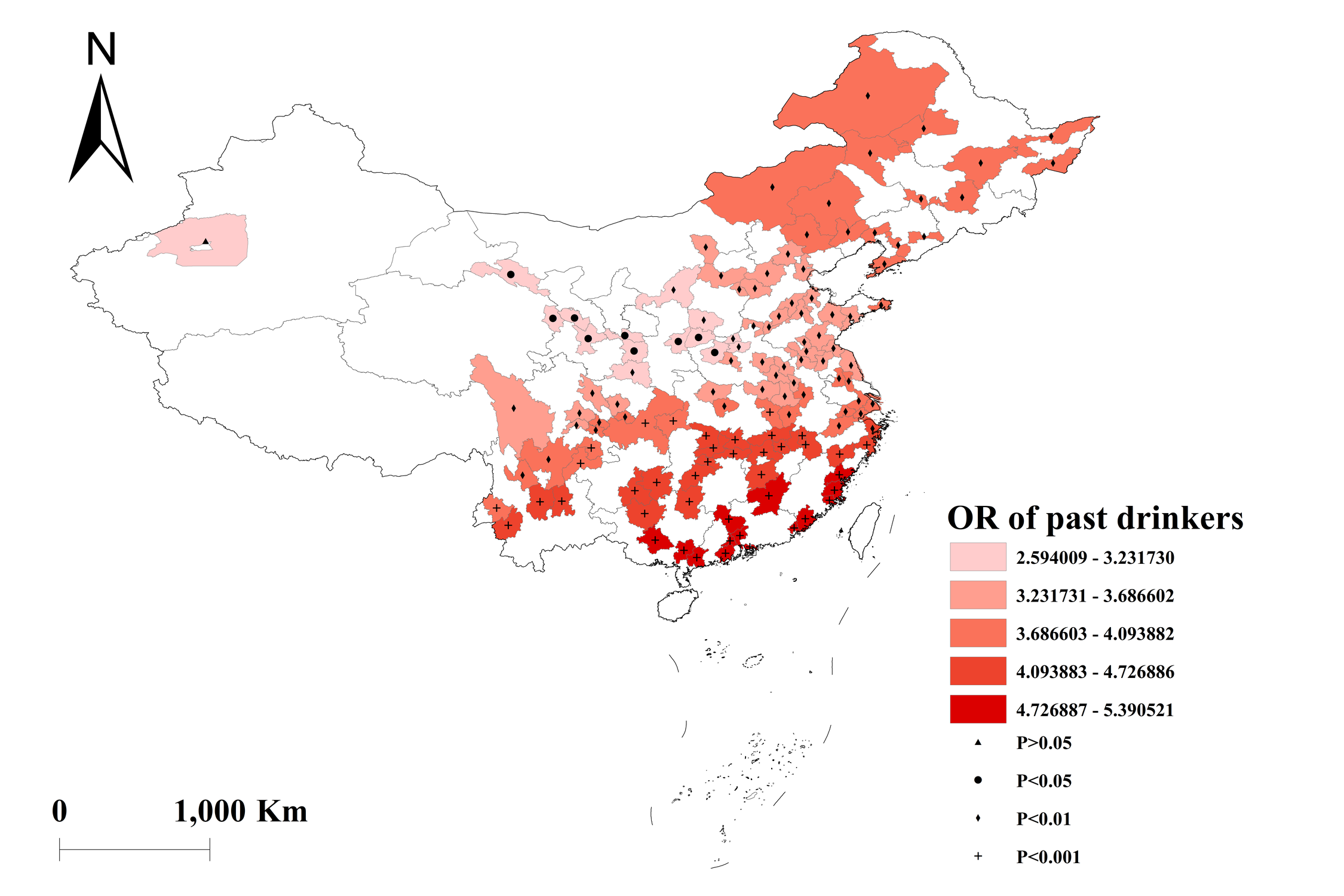

Supplement: S14 Fig — (TIF) [file pone.0286401.s014.tif]

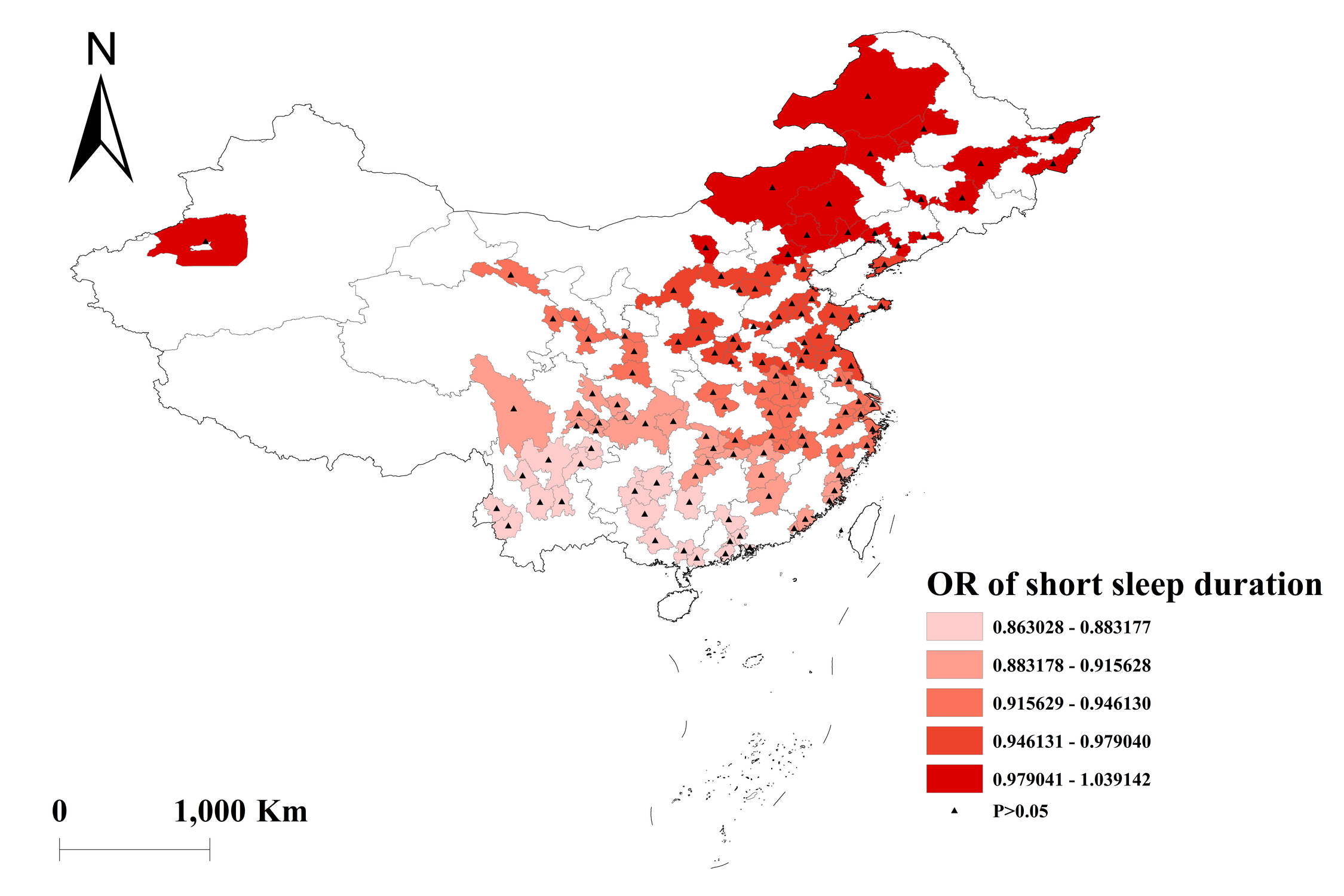

Supplement: S15 Fig — (TIF) [file pone.0286401.s015.tif]

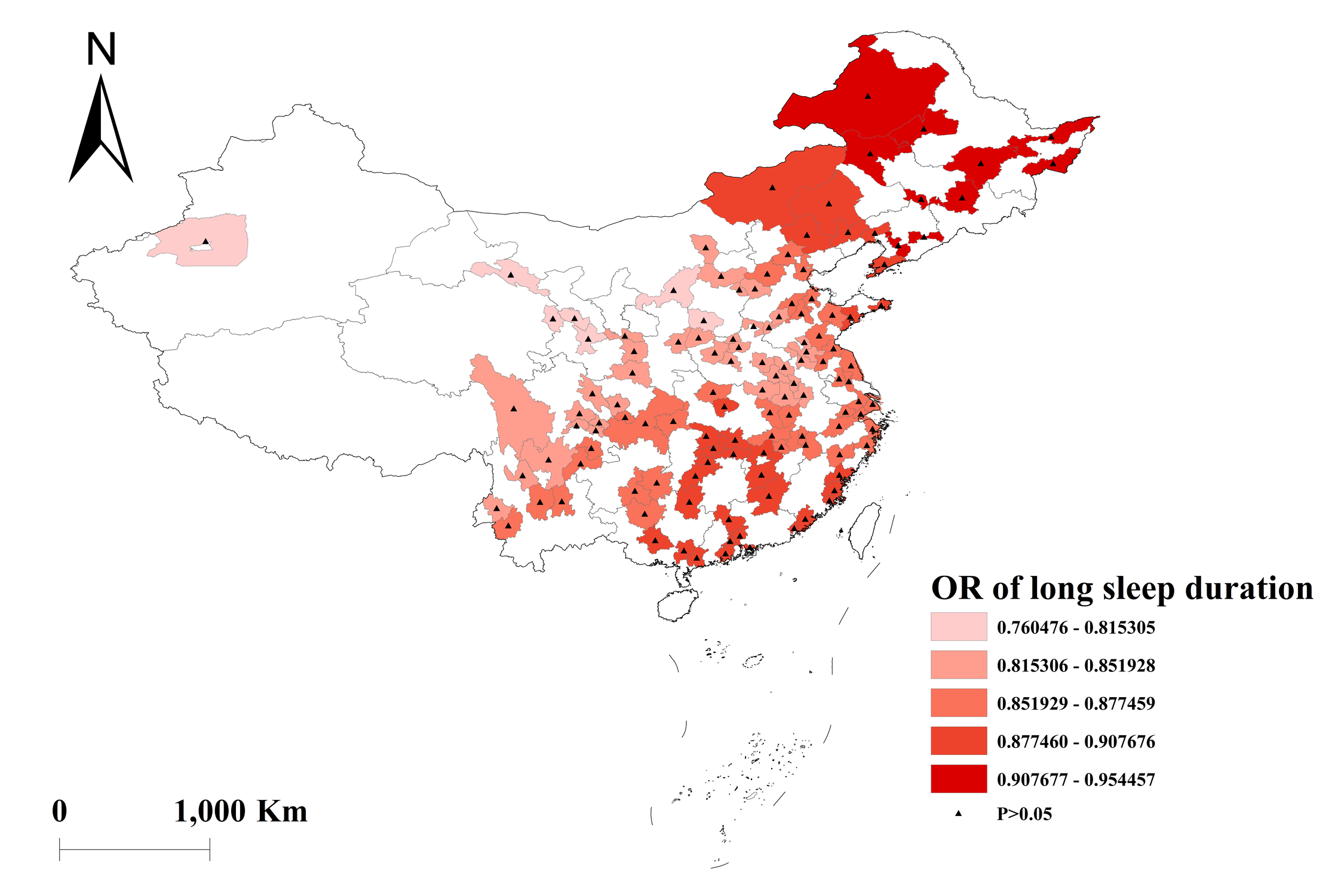

Supplement: S16 Fig — (TIF) [file pone.0286401.s016.tif]

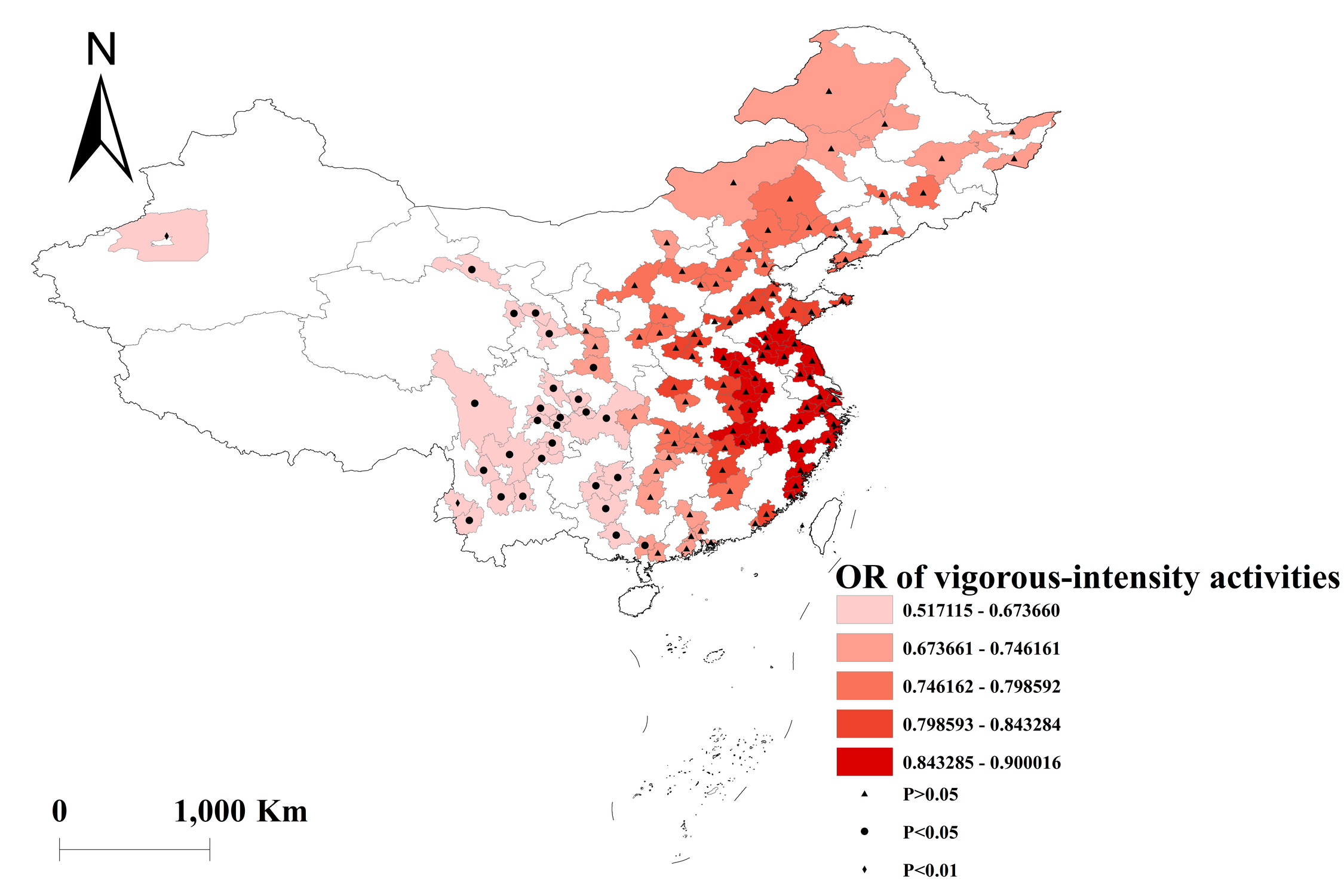

Supplement: S17 Fig — (TIF) [file pone.0286401.s017.tif]

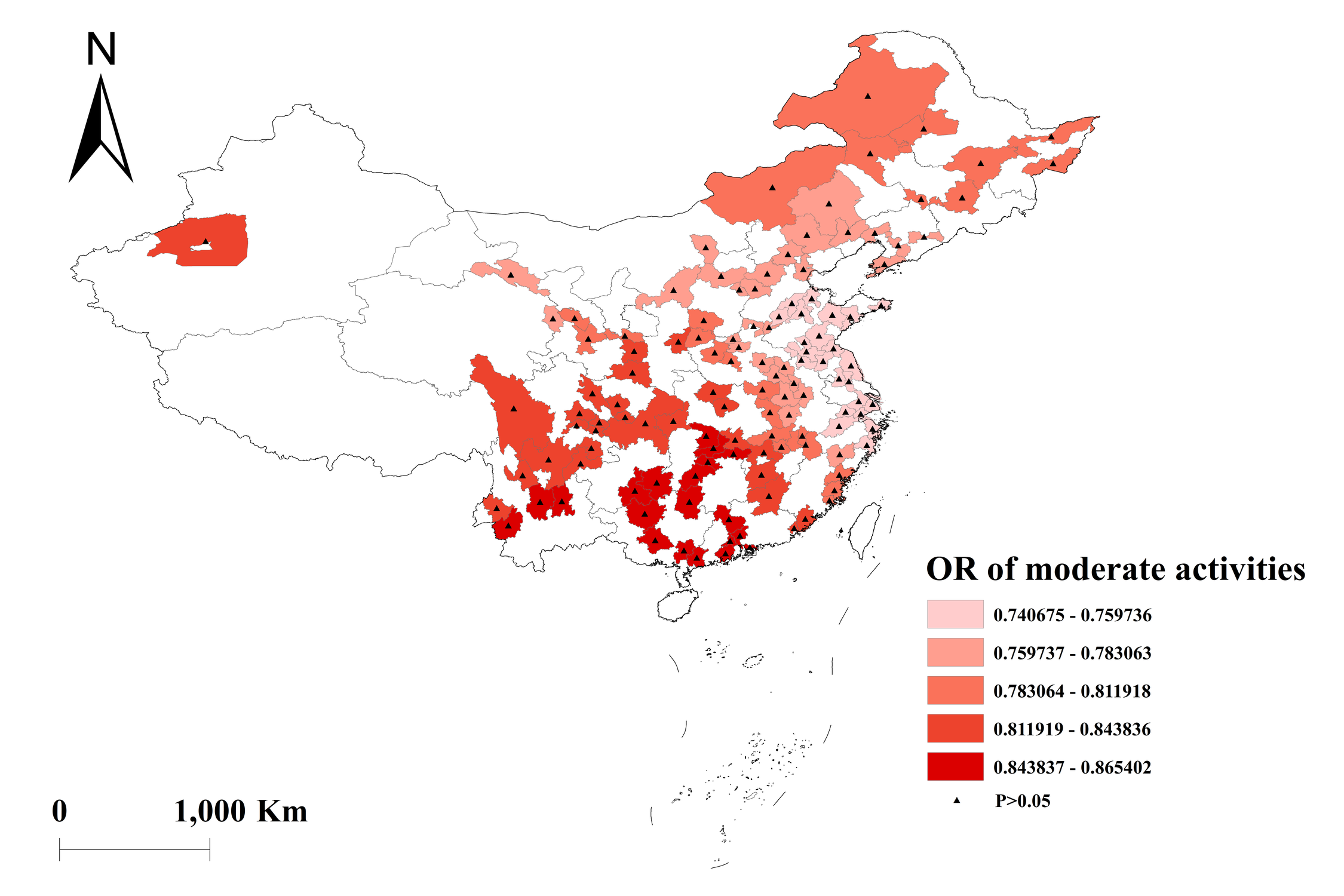

Supplement: S18 Fig — (TIF) [file pone.0286401.s018.tif]

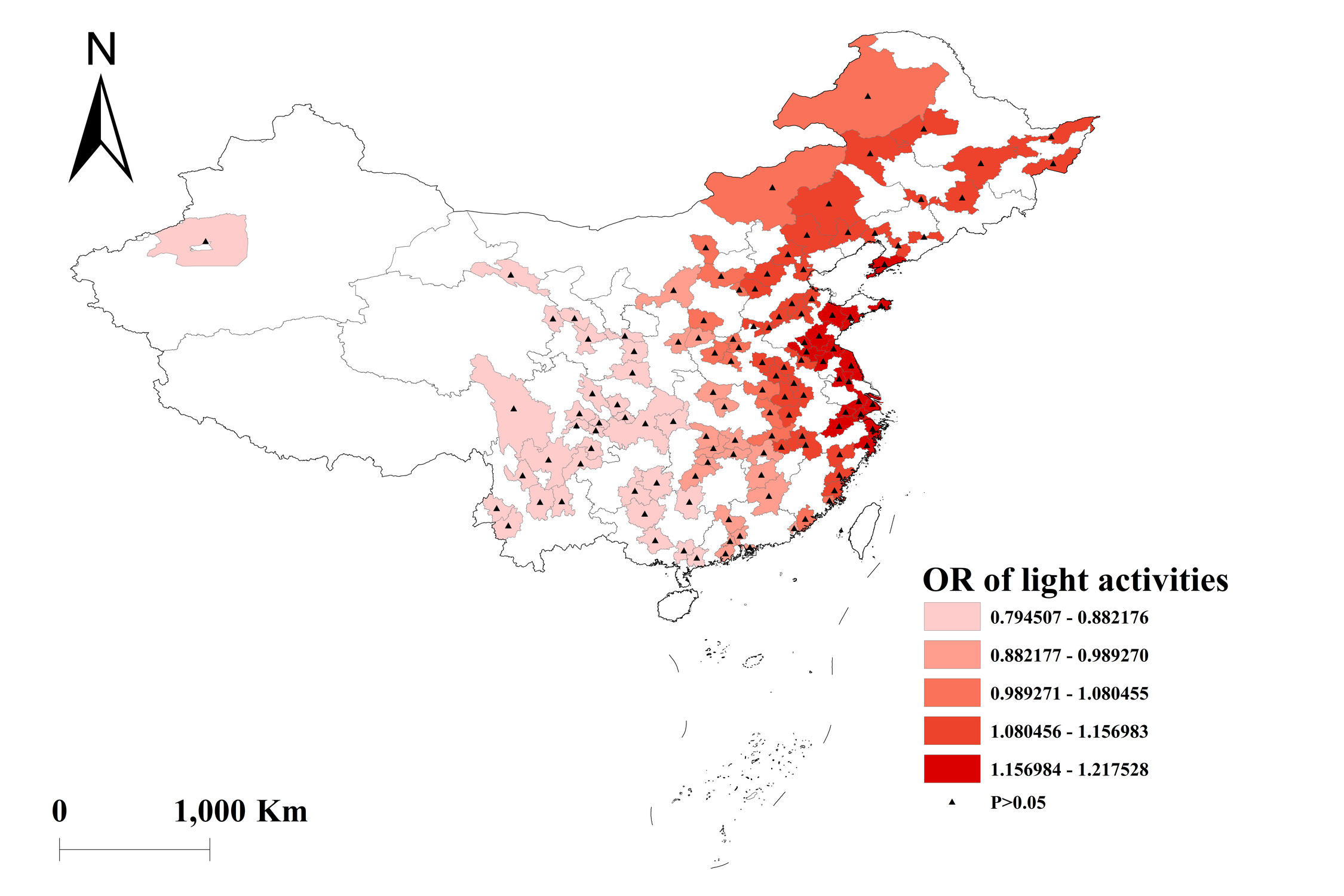

Supplement: S19 Fig — (TIF) [file pone.0286401.s019.tif]

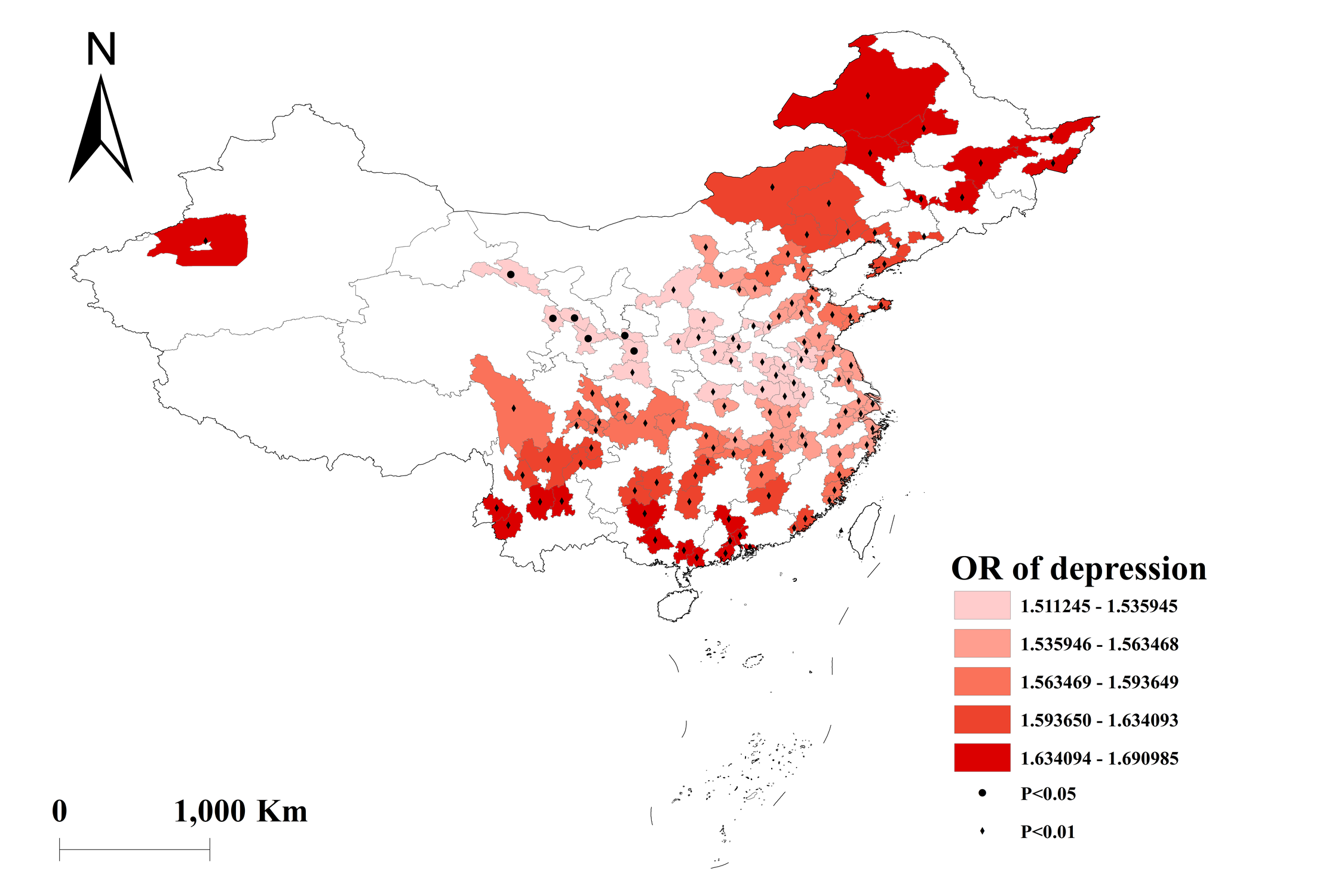

Supplement: S20 Fig — (TIF) [file pone.0286401.s020.tif]
