# Supplementary material for: Geographical specific association between lifestyles and multimorbidity among adults in China
Source: PLoS One. 2023 Jun 7;18(6):e0286401. doi: 10.1371/journal.pone.0286401 (PMC10246811; doi:10.1371/journal.pone.0286401)
Supplement: S1 File — (DOCX) [file pone.0286401.s021.docx]

**Materials and methods**

**Study variables**

The outcome variable of the study was multimorbidity, defined as the co-occurrence of at least two chronic diseases, including hypertension, diabetes or high blood sugar, heart disease (heart attack, coronary heart disease, angina, congestive heart failure, or other heart problems), and stroke, which were diagnosed by asking “Have you been diagnosed by a doctor with the conditions, including all chronic diseases listed above?”.

Sociodemographic factors and lifestyles were selected from the CHARLS. Sociodemographic characteristics containing age and gender were used as adjustment variables. The participants’ age was calculated from the survey time and birth date and was divided into 4 groups, 45-54, 55-64, 65-74, and 75 years old and above.

In this study, lifestyles included smoking, drinking, sleep duration, physical activities, and depression. Smoking was further divided into three subgroups for analyses, including current smokers (participants who currently smoke and have smoked more than 100 cigarettes), former smokers (participants who have now quit smoking but have smoked more than 100 cigarettes in the past), and non-smokers (participants who at the survey stated that they have never smoked) [1]. The drinking was classified as current drinkers (participants who drink more than once a month), past drinkers (participants who used to drink more than once a month but currently do not drink), and non-drinkers (participants who drink less than once a month). Sleep duration was categorized based on the National Sleep Foundation's recommendations for adults into three groups: short sleep duration (<6 h), normal sleep duration (6-8 h), and long sleep duration (>8 h) [2].

For physical activities were determined three levels based on activity intensity: incorporating vigorous-intensity, moderate and light activities. Vigorous-intensity activities can cause shortness of breath, such as carrying heavy stuff, digging, hoeing, aerobic workout, bicycling at a fast speed, riding a cargo bike/motorcycle, etc. Moderate activities can make you breathe faster than usual, such as carrying light stuff, bicycling at a normal speed, mopping, Tai-Chi, and speed walking. Light activities included walking (walking from one place to another place at a workplace or home, and taking a walk for leisure, sports, exercise or entertainment). All exercise was required to be done for at least ten minutes each time.

The short version of the Center for Epidemiological Studies Depression Scale (CES-D10), was used to determine whether the participants in the study were experiencing the depressive symptoms [3]. The CES-D10 consists of 10 items, each scored from 0 to 3, with higher score indicating more severe depression. The respondents were asked about their feelings and behaviors in the past week, and those with a total score of 10 or more were considered to have depressive symptoms. For this study, participants with a score of 0-9 were considered normal and vice versa.

**References**

1. He Y, Jiang B, Wang J, Feng K, Chang Q, Fan L, et al. Prevalence of the metabolic syndrome and its relation to cardiovascular disease in an elderly Chinese population. J Am Coll Cardiol. 2006;47(8):1588-94. doi: 10.1016/j.jacc.2005.11.074. PubMed PMID: WOS:000236819000012.

2. Ohayon M, Wickwire EM, Hirshkowitz M, Albert SM, Avidan A, Daly FJ, et al. National Sleep Foundation's sleep quality recommendations: first report. Sleep Health. 2017;3(1):6-19. doi: 10.1016/j.sleh.2016.11.006. PubMed PMID: WOS:000425610600003.

3. Lin FH, Yih DN, Shih FM, Chu CM. Effect of social support and health education on depression scale scores of chronic stroke patients. Medicine (Baltimore). 2019;98(44):6. doi: 10.1097/md.0000000000017667. PubMed PMID: WOS:000497731800040.
